# Glucocorticoids—All-Rounders Tackling the Versatile Players of the Immune System

**DOI:** 10.3389/fimmu.2019.01744

**Published:** 2019-07-24

**Authors:** Cindy Strehl, Lisa Ehlers, Timo Gaber, Frank Buttgereit

**Affiliations:** ^1^Department of Rheumatology and Clinical Immunology, Charité—Universitätsmedizin Berlin, Berlin, Germany; ^2^German Rheumatism Research Centre (DRFZ) Berlin, Berlin, Germany

**Keywords:** glucocorticoids, immune system, inflammation, giant cell arteritis, rheumatoid arthritis, systemic lupus erythematosus, allergic diseases, leukemia

## Abstract

Glucocorticoids regulate fundamental processes of the human body and control cellular functions such as cell metabolism, growth, differentiation, and apoptosis. Moreover, endogenous glucocorticoids link the endocrine and immune system and ensure the correct function of inflammatory events during tissue repair, regeneration, and pathogen elimination via genomic and rapid non-genomic pathways. Due to their strong immunosuppressive, anti-inflammatory and anti-allergic effects on immune cells, tissues and organs, glucocorticoids significantly improve the quality of life of many patients suffering from diseases caused by a dysregulated immune system. Despite the multitude and seriousness of glucocorticoid-related adverse events including diabetes mellitus, osteoporosis and infections, these agents remain indispensable, representing the most powerful, and cost-effective drugs in the treatment of a wide range of rheumatic diseases. These include rheumatoid arthritis, vasculitis, and connective tissue diseases, as well as many other pathological conditions of the immune system. Depending on the therapeutically affected cell type, glucocorticoid actions strongly vary among different diseases. While immune responses always represent complex reactions involving different cells and cellular processes, specific immune cell populations with key responsibilities driving the pathological mechanisms can be identified for certain autoimmune diseases. In this review, we will focus on the mechanisms of action of glucocorticoids on various leukocyte populations, exemplarily portraying different autoimmune diseases as heterogeneous targets of glucocorticoid actions: (i) Abnormalities in the innate immune response play a crucial role in the initiation and perpetuation of giant cell arteritis (GCA). (ii) Specific types of CD4+ T helper (Th) lymphocytes, namely Th1 and Th17 cells, represent important players in the establishment and course of rheumatoid arthritis (RA), whereas (iii) B cells have emerged as central players in systemic lupus erythematosus (SLE). (iv) Allergic reactions are mainly triggered by several different cytokines released by activated Th2 lymphocytes. Using these examples, we aim to illustrate the versatile modulating effects of glucocorticoids on the immune system. In contrast, in the treatment of lymphoproliferative disorders the pro-apoptotic action of glucocorticoids prevails, but their mechanisms differ depending on the type of cancer. Therefore, we will also give a brief insight into the current knowledge of the mode of glucocorticoid action in oncological treatment focusing on leukemia.

## Introduction

Hormones enable intercellular communication as well as the exchange of information between different organ systems throughout the human body. They are involved in a variety of processes such as growth, development, and metabolism. The synthesis and secretion of hormones is subject to stringent regulations, comprising positive, and negative feedback loops as crucial mechanisms. Steroids are lipophilic hormones that are subdivided into mineralocorticoids produced in the zona glomerulosa of the adrenal cortex, glucocorticoids produced in the zona fasciculata as well as sex hormones produced in the zona reticularis and to a great extent in the gonads. Since it has been demonstrated that natural glucocorticoids also have some mineralocorticoid effects, the classification into these groups is not completely accurate. The term “glucocorticoids” is more suitable when talking about synthetic glucocorticoids (e.g., prednisolone or dexamethasone), because these drugs are more restricted to glucocorticoid effects only (1).

The initial step of steroid hormone biosynthesis is the conversion of cholesterol to the precursor pregnenolone in the mitochondria. Steroid hormone biosynthesis is mainly realized by enzymes of the cytochrome P450 family (2). Sex hormones affect growth, development and reproductive cycles whereas mineralocorticoids regulate sodium and water balance and glucocorticoids influence energy and metabolic processes as well as immune and stress responses.

Between 5 and 30 mg of the active endogenous (physiological) glucocorticoid cortisol is produced per day, regulated by the hypothalamic–pituitary–adrenal (HPA) axis. Glucocorticoids bind to glucocorticoid receptors that are present in cells throughout the body, including cells in the hypothalamus and pituitary gland, which are part of the negative feedback loop controlling the glucocorticoid production. Furthermore, the hormone concentration varies in a circadian manner peaking at 9 a.m. in the morning and reaching the lowest plasma concentration at midnight.

The dehydrogenation of cortisol to its inactive form cortisone is promoted by the enzyme 11β-hydroxysteroid dehydrogenase (11β-HSD) type 1 in the liver. The same enzyme also exhibits reductase activity promoting the reverse reaction. The type 2 11β-HSD is only able to convert the active into the inactive form due to its sole dehydrogenase activity. Depending on the balance and activity of both enzymes, the intracellular glucocorticoid concentration and thus the tissue sensitivity for glucocorticoids varies (3). In addition to that, glucocorticoids have been demonstrated to possess immunomodulating effects which depend on concentration and time of administration: While an immunostimulatory effect is observed at lower concentrations (below serum level), higher concentrations (therapeutic range) lead to an immunosuppression (4). Due to their strong immunosuppressive, anti-inflammatory and anti-allergic effects, synthetic glucocorticoids have been established as important drugs in the treatment of diseases driven by immune and inflammatory dysregulation.

### Glucocorticoid Signaling

Glucocorticoids are lipophilic substances with a low molecular weight that can easily pass cellular membranes and bind to the glucocorticoid receptor in the cytosol. The cytosolic glucocorticoid receptor is ubiquitously expressed by nucleated cells and resides in the cytoplasm as a multi-protein complex. Proteins and co-factors stabilize the receptor and support a specific conformation leading to a high binding affinity for its ligands (5–9). Two main receptor isoforms are described, the α glucocorticoid receptor, which is activated by glucocorticoids, and the β isoform with a deformed ligand-binding domain that cannot bind ligands (10–13). Further receptor isoforms which differ in their transcriptional activity as a result of alternative splicing and/or post-translational modifications, have been extensively described elsewhere (12–14).

The hormone-receptor complex is translocated into the nucleus as a homodimer and binds to palindromic DNA-binding sites in the promoter region of different target genes, so called glucocorticoid response elements. This genomic mechanism of glucocorticoid action is known as transactivation, which describes the binding to positive glucocorticoid response elements leading to the activation of the transcription of anti-inflammatory but also regulatory proteins. These include for example IL-10, Annexin 1, and IκB as well as enzymes of gluconeogenesis such as tyrosine aminotransferase, serine dehydrogenase, or phosphoenol pyruvate carboxykinase. In contrast, the term transrepression refers to an impairment of the expression of immunoregulatory and proinflammatory proteins caused by (i) competition for nuclear co-activators between the hormone-receptor-complex and transcription factors; (ii) direct or indirect interaction with transcription factors like NF-κB and AP-1. Similarly, glucocorticoids diminish gene expression by a mechanism referred to as cis-repression, which involves binding to negative glucocorticoid response elements. Genomic mechanisms of glucocorticoid action result in “delayed effects,” meaning that the protein level does not change directly after glucocorticoid administration. The duration of the delay depends on different factors, including transport within the bloodstream, onset of activation/translocation of the hormone-receptor complex and the transcriptional and translational processes themselves. Nevertheless, the description of rapid improvements which are observed within a few minutes—especially after intravenous or intraarticular injection of high glucocorticoid doses—demonstrates the existence of non-genomic effects. These are triggered by (i) proteins released from the multi-protein complex after the binding of glucocorticoids to the cytosolic receptor, (ii) interactions with membrane-bound receptors, and (iii) nonspecific effects resulting from the interaction of glucocorticoids with cellular membranes (15, 16).

More pronounced glucocorticoid effects are observed with increasing glucocorticoid dosages, as receptor saturation is achieved (17). Unfortunately, rising dosages and duration of administration simultaneously increase the risk of adverse events. While the long-term use of dosages ≤ 5 mg prednisone equivalent per day is generally associated with a low risk of adverse effects, the application of dosages >10 mg/day increases the frequency of the latter (18). These adverse effects are thought to depend on the mechanism of glucocorticoid action: Repression of cytokines such as IL-1, IL-2, IL-6, TNF-α, IFN-γ, and prostaglandins mediates the positive anti-inflammatory effects, while transactivation is thought to be responsible for the majority of adverse effects (19–21). However, this classification is not absolute. In contrast, it has been demonstrated that transactivation also contributes to the anti-inflammatory effects, e.g., by the upregulation of genes like GILZ and DUSP1. In addition, in a mouse model the prevention of receptor dimerization and thereby inhibition of DNA-binding impaired the anti-inflammatory capacity of glucocorticoid action (22–26).

### Glucocorticoids and Inflammation

These findings clearly show that our knowledge concerning the mechanisms of glucocorticoid action—including the desirable anti-inflammatory and the undesirable adverse effects—is yet insufficient. Nevertheless, these drugs still represent an indispensable component of the treatment of most inflammatory diseases because of their efficient and cost-effective characteristics. However, the considerable toll taken by adverse events must not be neglected and the development of an equally effective alternative with a more favorable side-effect profile would be most desirable. The extent and importance of glucocorticoid toxicity has been reviewed elsewhere (27, 28) and will not be discussed in detail in this article.

**Figure 1:**
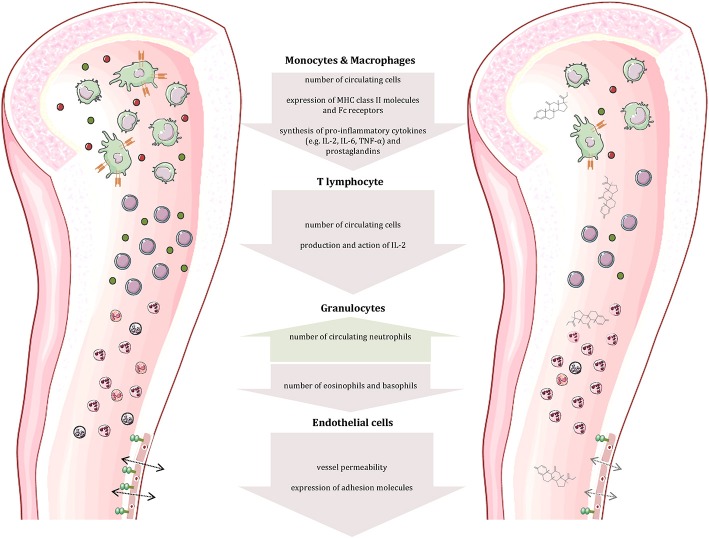
Effects of glucocorticoids on immune and other cells. Glucocorticoids affect the number and function of immune cells (cells and compartments adapted from Servier Medical Art, 2007; Les Laboratoires Servier, München, Germany).

The immune system consists of two major components: The innate immune response represents our first line of defense and includes physical and chemical barriers such as the skin and tears. In addition, non-specialized cells recognize foreign invaders by components like bacterial lipopolysaccharide and destroy them by phagocytosis or release of toxic substances. The adaptive immune response—our second line of defense—includes B and T lymphocytes. While the former are responsible for antibody production, the latter can differentiate into distinct subpopulations that participate in B cell maturation or possess cytotoxic potential (29–31). The two lines of defense are linked by cytokines and cell-cell interaction, which is crucial for the initiation of the adaptive response. The most notable attribute of the adaptive immune response is memory, enabling an immediate and very specific pathogen defense following previous exposure. The protective actions of the immune system are accompanied by pain, swelling, itching, redness and heat, typical signs of an inflammation. At the same time, these symptoms represent a significant burden in autoimmune diseases. Normally, the immune response is strictly regulated to discriminate self from non-self—a mechanism known as tolerance (29, 30). It is realized by positive and negative selection of lymphocytes in the bone marrow or thymus. In more detail (for T cells), T cells that cannot bind MHC class 1 or class 2 complexes undergo apoptosis due to the lack of survival signals. The subsequent negative selection determines if T cells bind self-peptides presented by epithelial cells of the thymus. Naive T cells that have passed both, the positive and the negative selection are qualified to migrate into secondary lymphoid organs (29). Autoimmune diseases originate from a dysregulation of the immune response, while the particular cause of the disease is often unknown. Some factors, including genetic predisposition, sex, and environment have been identified to promote the establishment of autoimmune diseases. Due to their strong anti-inflammatory and immunosuppressive effects on almost all immune cells (summarized in Figure 1), glucocorticoids are indispensable in the treatment of autoimmune diseases. In general, glucocorticoids inhibit leukocyte traffic and thereby the access of leukocytes to the site of inflammation. Furthermore, glucocorticoids interfere with immune cell function and suppress the production and actions of humoral factors involved in the inflammatory process.

Since the establishment and the course of autoimmune diseases are driven by different cell populations, glucocorticoid application targets diverse leukocyte populations and thus the mechanism of glucocorticoid action varies. Recently, Franco et al. have investigated the transcriptional effects of glucocorticoids on nine primary human cell types. They found 9,457 genes to be differentially expressed in response to glucocorticoids, whereas only 25 of them (0.3%) involved all cell types examined, demonstrating that the transcriptional response of each cell type is quite distinct (32).

The next chapters will illustrate the versatile modulating effects of glucocorticoids on the immune system on the basis of exemplary diseases involving the respective leukocyte population. Glucocorticoid regimens used in daily practice according to current guidelines are presented in Table 1 for the selected diseases.

**Table 1:** Glucocorticoid regimens in selected diseases.

**Disease**	**Induction**	**Tapering**	**Maintenance**	**Relapse**
**INFLAMMATORY RHEUMATIC DISEASES**				
Giant cell arteritis	- Immediate treatment with 40–60 mg/day^*^ for induction of remission in active GCA^**^ (33)	- Tapering is recommended when the disease is under control to achieve a target dose of 15–20 mg/day^*^ within 2 to 3 months - After 1 year target dose should be ≤ 5 mg/day^*^ (33)	- If long-term therapy is required a dose of 5 mg/day^*^ or less should be used - GC therapy should ideally be tapered to zero as early as clinically feasibly (18)	- Increase to pre-relapse dose or by up to 5–10 mg/day^*^ - Taper within 4–8 weeks to pre-relapse dose - Repeat induction therapy for ischemic complications (34)
Rheumatoid arthritis	- When initiating/changing csDMARDs short-term GC therapy should be considered (35)	- GC tapering should start as soon as clinically feasible (35)	- If long-term therapy is required a dose of 5 mg/day^*^ or less should be used - GC therapy should ideally be tapered to zero as early as clinically feasibly (18)	- Usually doses between 10 and 20 mg/day^*^ are sufficient to treat flares in this disease
Systemic lupus erythematosus	- Therapy depends on disease manifestations and severity (36) - In acute, organ-threatening disease high-dose intravenous pulse therapy (usually 250–1,000 mg/day^*^ for 3 days) is often used (36)	- GC should be tapered or at least minimized as rapidly as clinically feasible	- Long-term aim is to minimize daily dose to ≤ 7.5 mg/day^*^ or to discontinue GC therapy (36)	- The characteristic of flare therapy depends on disease, as has been similarly stated for the induction therapy
**ATOPY**				
Atopic dermatitis	- Stepwise approach: adjust treatment based on disease severity assessed by SCORAD (37)			
	→ Mild disease: class II topical glucocorticoids (e.g., flumethasone 0.02%) (38)			
	→ Moderate disease: class II/III topical glucocorticoids (e.g., mometasone 0.1%) (39)			
	→ Severe disease: short-term oral glucocorticoids may be considered in adults (38)			
Allergic rhinitis	- Moderate to severe rhinitis: nasal glucocorticoids, e.g., fluticasone, mometasone, beclametasone (40, 41)			
	- Oral glucocorticoids should only be used in severe persisting disease (40, 41)			
	- stepped-care approach according to disease severity (42)			
Asthma	- Most patients initially receive low dose ICS (e.g., 200–400 μg/d budesonide) (43) - Frequent troublesome symptoms justify medium (400–800 μg/d) to high dose ICS (>800 μg/d) (44) - Low dose oral corticosteroids ( ≤ 7.5 mg/day ^*^) should only be considered in adults with severe asthma or poor symptom control (45)	- ICS should not be stopped completely, cessation is associated with a higher risk of exacerbations (46) - In stable disease ICS doses can be reduced by 25–50% every 3 months (47)	- ICS are recommended as controller treatment in all asthma patients either as-needed or daily depending on disease severity (43) - Dose adjustment according to a stepwise approach^***^ ranging from 200–400 to >800 μg/d budesonide or comparable doses of other formulations in adults, reduced doses are used in the treatment of children <12 years (48)	- *Worsening symptoms:* adjustment of the treatment (increase reliever/controller use, step up to higher dose) according to a written asthma action plan^***^ - Severe exacerbation: → adults: 40–50 mg/d prednisolone → Children: 1–2 mg/kg/d, max. 40 mg/d prednisolone to be continued for 5–7 days (49, 50)
Anaphylactic shock	- Glucocorticoids are used to prevent protracted anaphylactic symptoms, while their efficacy in the acute phase is limited due to slow onset of action (51, 52)			
	−250–1,000 mg i.v. prednisolone (weight-adjusted dosing in children) (53)			
**LEUKEMIA**				
Chronic lymphoblastic leukemia^****^	- Patients with diagnosed limited-stage Hodgkin's lymphoma (HL) and a positive interim positron-emission tomography after two cycles of ABVD (adriamycin, bleomycin, vinblastine, and dacarbazine) should be treated with two cycles of bleomycin/etoposide/doxorubicin/cyclophosphamide/vincristine/procarbazine/prednisone in escalated dose before ISRT			
	- Patients with refractory or relapsed HL dexamethasone can be given in combination with high-dose cytarabine/cisplatin (DHAP) before high-dose chemotherapy followed by autologous stem cell therapy			
	- Patients diagnosed for nodular lymphocyte predominant Hodgkin lymphoma benefit from the combination of rituximab/cyclophosphamide/doxorubicin/vincristine/prednisone (R-CHOP)			
	- CLL patients with transformation into a diffuse large B-cell lymphoma benefit from therapies used in DLBCL such as rituximab plus CHOP (cyclophosphamide, vincristine, doxorubicin, and dexamethasone) (54–56)			
Chronic myeloid leukemia	N/A			
Acute myeloid leukemia	N/A			
Acute lymphoblastic leukemia	- Glucocorticoids are given as a so-called pre-phase therapy (usually prednisone 20–60 mg/day or dexamethasone 6–16 mg/day, both *i.v*. or *p.o*.) alone, or in combination with another drug (e.g., vincristine, cyclophosphamide), but often given together with allopurinol and hydration for ~5–7 days. The response to pre-phase therapy defines the chemosensitivity of the disease, and is included in some studies for risk assessment, since good responders to prednisone may have a better outcome.			
	- Regimens of induction therapy are centered on vincristine, glucocorticoids, and anthracycline (daunorubicin, doxorubicin, rubidazone, idarubicin), with or without cyclophosphamide or cytarabine. Dexamethasone is often preferred to prednisone, since it penetrates the blood–brain barrier and also acts on resting leukemic blast cells (LBCs).			
	- In adult ALL glucocorticoids are often used in the hyper-CVAD (cyclophosphamide, vincristine, doxorubicin, dexamethasone) protocol, preferentially used in the United States, but also in other parts of the world			
	- Maintenance therapy usually consists of daily 6-mercaptopurine and weekly methotrexate. In some treatment regimens, repeated cycles of vincristine, dexamethasone or other drugs in monthly or longer intervals are given (57)			

* *Doses are given as prednisone-equivalent*.

** *In patients with GCA suffering from acute visual loss or amaurosis fugax, the use of very high GC dosages, namely 0.25–1 g i.v. methylprednisolone daily for up to 3 days should be considered*.

*** *Details are provided by the Global Initiative for Asthma. Global Strategy for Asthma Management and Prevention, 2019. Available from: www.ginasthma.org N/A: glucocorticoids are not used as standard therapy in these diseases*.

**** *The transformation into a diffuse large B-cell lymphoma (DLBCL) or Hodgkin's lymphoma occurs in 2%−15% of CLL patients during the course of their disease*.

## Abnormalities in Innate Immune Response Play a Crucial Role in the Initiation and the Perpetuation of Giant Cell Arteritis

**Figure 2:**
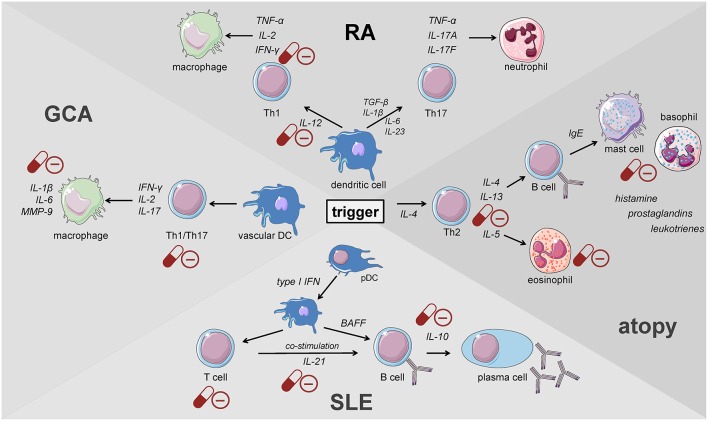
Key players of the immune system driving the pathogenesis of immune-mediated diseases. GCA, giant cell arteritis; DC, dendritic cell; pDC, plasmacytoid dendritic cell; RA, rheumatoid arthritis; SLE, systemic lupus erythematosus (cells adapted from Servier Medical Art, 2007; Les Laboratoires Servier, München, Germany).

Giant cell arteritis (GCA) is defined as a granulomatous large-vessel vasculitis, which primarily involves medium- and large-caliber branches of the aorta (34, 58, 59). Both the innate and the adaptive immune responses are involved in the pathogenesis of this disease which can be divided into different phases (59). The initiation of inflammation is followed by its amplification and the constitution of feed forward loops leading to arterial remodeling and ultimately vascular damage. Recently, the current knowledge on the pathophysiology of GCA has been discussed in detail in two reviews (59, 60). Al-Mousawi et al. describe this disease as being mainly mediated by T cells (60). The first step, however, is the abnormal maturation of vascular dendritic cells (DC) in the adventitia of the affected vessels. An unknown trigger, perhaps microorganisms or viral agents, drives this initial step (59). Predisposing factors include a certain genetic background, female sex, and alterations of the immune and arterial systems related to aging (59). The activated DC recruit and activate CD4+ naïve T cells in the arterial wall where they polarize into T helper (Th) 1 cells, Th17 and regulatory T (Treg) cells (59, 60). The secreted products of these cells, namely and most importantly interferon-γ, interleukin (IL)-2, and IL-17, facilitate both the recruitment and activation of neutrophils, macrophages and vascular smooth muscle cells, and the formation and activation of multinucleated giant cells (Figure 2). These giant cells are also capable of secreting cytokines and growth factors. Of note, Th17 cells also secrete other cytokines such as IL-21, IL-22, IL-8, and IL-26. Macrophages produce IL-6 and IL-1β within the adventitia. The latter cytokines are thought to mainly drive the systemic manifestations of GCA such as fatigue, fever, and weight loss. The fact that the levels of these cytokines largely determine glucocorticoid requirements underlines the importance of the innate immune response in the pathogenesis of GCA (61). Macrophages also produce matrix metalloproteinases (MMP) such as MMP-9, a type IV collagenase. Watanabe et al. have very recently identified this enzyme in vasculitic lesions of GCA and have shown MMP-9 to control the access of monocytes and T cells to the vascular wall. MMP-9–producing monocytes facilitate migration of T cells through the collagen IV-containing basement membrane. The enzymatic activity of MMP-9 is required for invasion of vasculitogenic T cells and monocytes, formation of neoangiogenic networks, and neointimal growth (62). As a consequence, the elastic lamina and growth factors are destroyed, which propagates intimal hyperplasia. Of note, macrophages also produce reactive oxygen species which contribute to the damage of smooth muscle cells in the media (60). Ultimately, the injured arterial cells respond to the damaging immunological events mentioned above by initiating dysfunctional repair processes. This vascular remodeling leads to inflammatory wall thickening, decreased luminal diameter, and ischemic manifestations of GCA with potential organ damage (34).

Glucocorticoids represent a most effective therapy and, therefore, remain—despite the recently shown favorable effects of the IL-6 receptor inhibitor Tocilizumab (63)—the primary treatment in GCA (34). These drugs have been the mainstay of treatment since the 1950s. Their genomic and non-genomic effects contribute to the successful treatment of this disease. We have recently summarized details regarding glucocorticoids in the management of polymyalgia rheumatica and GCA (64). In brief, glucocorticoids induce important anti-inflammatory and immunosuppressive effects on both primary and secondary immune cells involved in the pathophysiology as described above. Glucocorticoids inhibit some of their crucial functions with key mechanisms being the suppression of the production of pro-inflammatory cytokines, and the prevention and inhibition of activation of T cells and monocytes/macrophages.

**Figure 3:**
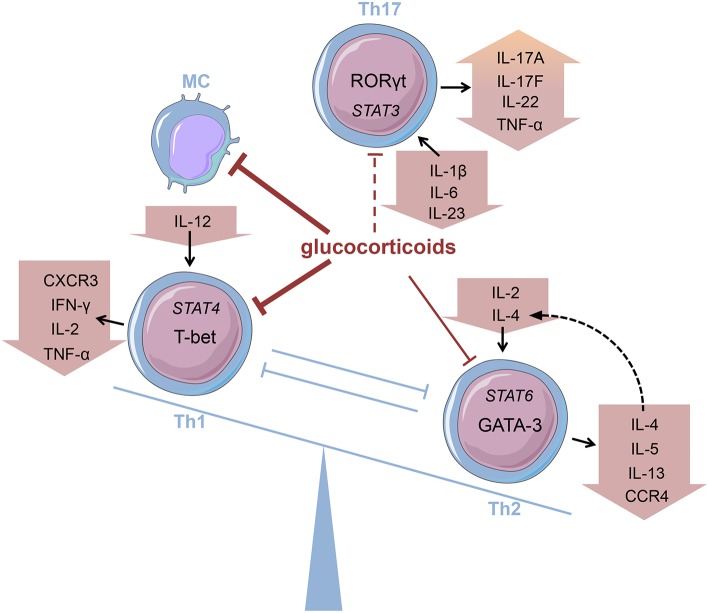
Glucocorticoids modifying the Th balance Glucocorticoids affect the predominance of different T helper (Th) cell subsets, e.g., by influencing cytokine production. MC, monocyte (cells adapted from Servier Medical Art, 2007; Les Laboratoires Servier, München, Germany).

Innate immune cells that are predominantly responsible for the features of systemic inflammation present in GCA are most susceptible to glucocorticoid treatment (65, 66). By inhibiting the NFκB pathway by direct or indirect interaction with this transcription factor as described in the introduction, glucocorticoids efficiently suppress the production of central cytokines (Figure 2) (67). In this context, Linden and Brattsand demonstrated that GM-CSF showed the highest susceptibility to glucocorticoid treatment compared to IL-1β and IL-6 (68). These findings conform to the beneficial effects of IL-6 blockade in GCA therapy (63). Of note, higher glucocorticoid sensitivity has been attributed to monocytes compared to more differentiated macrophages (68). Consequently, it can be inferred that glucocorticoids are most potent in inhibiting freshly attracted monocytes in states of acute inflammation. In addition, glucocorticoids affect the recruitment of cells of the mononuclear phagocytic system by suppressing the expression of adhesion molecules on the surface of the endothelium (69). With respect to monocyte function, Blotta et al. demonstrated that the incubation of monocytes with dexamethasone led to a decreased IL-12 production *in vitro* (Figure 3) (70). In line with this, they presented a limited capacity to induce Th1 differentiation.

Deng et al., however, have shown that glucocorticoids suppress the production of Th17-promoting cytokines (IL-1β, IL-6, and IL-23) (Figure 3), but IFN-γ-producing Th1 responses persist in treated patients (71). Also, patients presenting prominent expression of IL-17A in temporal artery biopsies demonstrated favorable responses to glucocorticoid treatment (72). Therefore, it was assumed that the IL-6-IL-17 cluster is highly responsive to glucocorticoid therapy, whereas the IL-12-IFN-γ cluster is resistant to glucocorticoid-mediated immunosuppression (73). Nevertheless, there are reports of a reduction in Th1 response after glucocorticoid treatment in patients with Takayasu's arteritis—a condition closely linked to GCA (74). Moreover, further studies revealed a decrease in both Th1 and Th17 cells, and a reduction of IFN-γ in GCA patients after glucocorticoid treatment (75, 76). Reviewing the pathogenesis of GCA, Samson et al. thus concluded that the conflicting results regarding glucocorticoid response result from prevalent plasticity between Th1 and Th17 cells influenced by the surrounding cytokine milieu (77).

At higher glucocorticoid dosages, for instance in form of pulse therapy in complicated GCA and in case of established visual loss, rapid non-genomic effects as already described in the introduction contribute to their therapeutic efficacy [reviewed in (64)].

## Autoimmune Diseases Driven by Irregularities in the Adaptive Immune System

### Th1 and Th17 Cells Represent Important Players in the Establishment and Course of Rheumatoid Arthritis

Rheumatoid arthritis (RA) is a systemic autoimmune disease that affects up to 1% of the population worldwide with a higher prevalence in women than in men. RA patients suffer from pain, immobility, and fatigue leading to decreased quality of life (78). The pathogenesis of RA is characterized by chronic inflammation mainly localized in the synovial joints leading to the destruction of articular cartilage and the establishment of bone erosions. Joint inflammation is accompanied by the infiltration of the synovium with immune cells such as T cells, B cells, macrophages, and dendritic cells and the proliferation of fibroblast-like synoviocytes of the synovial sub-lining layer which finally contribute to the joint destruction (79).

Glucocorticoids play a very important role in the treatment of RA, rapidly suppressing inflammatory activity especially at disease onset and during flares (15, 16, 80–82). Although glucocorticoids satisfactorily suppress inflammation and reduce symptoms such as pain and morning stiffness, data regarding their ability to manage cartilage degradation and bone erosions remain controversial (83, 84). Only limited success with regard to remission rates using glucocorticoids has been reported, e.g., in early treatment of undifferentiated arthritis (85) but also the SAVE trial (remission-rate: 17%) (86) and the STIVEA trial (remission-rate: 20%) (87). However, glucocorticoids still efficiently limit inflammation. Although the exact mechanism of RA pathogenesis remains unclear, it has become evident that Th cell subsets play an important role in the course of the disease. CD4+ T cells, especially Th1 and Th17 cells, play a major role in RA (88). RA patients present an enrichment of effector memory CD4+CD45RO+ T cells in the affected joints (89) and a massive expansion CD4+ T cell clones in synovial tissue of early disease, which suggests a local antigen-induced proliferation (90). In this context, it should be noted that blocking co-stimulation targeting CD80/CD86-CD28 interaction significantly improved the signs and symptoms of RA illustrating the importance of T cells in the pathogenesis (91). Moreover, genetic association of certain HLA-DRB1 alleles with increased susceptibility to RA further supports the central role of Th cells in RA (92).

When the Th1/Th2 paradigm dominated the understanding of the pathogenesis of autoimmune diseases, RA was defined as a Th1-driven disease because CD4+ T cells identified to be enriched in synovial fluids from RA patients were prone to secrete IFN-γ but not IL-4 (93, 94). These findings were further supported by the enrichment of the IFN-γ-induced chemokines CXCL9 and CXCL10 and the chemokine receptor CXCR3 binding both of the latter in RA synovium (95–98). Th1 cells classically activate macrophages and increase their capacity to produce pro-inflammatory cytokines present in RA synovium such as TNF (Figure 2) (99). Moreover, IL-12, IL-18, and IFN-γ, the drivers of Th1 differentiation have been also identified in synovial tissues of RA patients (100, 101), although the levels of Th1-mediated IFN-γ were relatively low compared with those of TNF-α, IL-1, or IL-6 derived from synovial fibroblasts (102, 103).

The discovery of Th17 cells (100, 101) and the delineation of the IL-17 family members (104) as well as the shift from Th17 cells to Th1 cells (i.e., “non-classic Th1 cells”) being more pathogenic than Th17 cells *per se* shed new light on the contribution of inflammatory Th subsets to the initiation of RA (Figure 2) (105–108). Th17 cells are highly unstable and easily shift to Th1 cells but can also transdifferentiate back as demonstrated for Th1 cells in the gut (109–112). At the onset and in the early phase of the pathogenesis of RA, Th17 cells shift to Th1 cells, whereas methotrexate (MTX) reduced the ratio of Th17 cells but not Th1 cells (113). Finally, these finding demonstrate that Th17 and ex-Th17 or “non-classic Th1 cells” cells play important roles in the early phase of RA and for the treatment using a combination of MTX and glucocorticoids according to the EULAR recommendations for the management of rheumatoid arthritis (35). While MTX reduces the ratio of Th17 cells, which are—depending on the immunopathological setting—resistant to glucocorticoid mediated suppression in terms of survival and the production of IL-17A and IL-17F but not IL-22 (114), glucocorticoids induce Th1 cell apoptosis via induction of BIM (114, 115). Moreover, glucocorticoids decrease IFN-γ production by T cells from patients with rheumatoid arthritis *ex vivo* and *in vitro* mechanistically via their suppressive action on the IL-12-induced STAT4 phosphorylation and by direct protein-protein interaction with the transcription factor T-BET—described as transrepression in the introduction (Figure 3) (116–119). Inhibition of Th1 activity by glucocorticoids may reduce overall inflammation in RA patients while the glucocorticoid resistant joint destruction can be assumed to be Th17 mediated. Mechanistically, glucocorticoid resistant joint destruction may be maintained by the glucocorticoid-mediated promotion of intrinsic Th17 differentiation (120), and the induction of bone resorption via synovial IL-17 (121). IL-17 also contributes to neutrophil recruitment (122) and an increase in neutrophil survival, a hallmark of RA synovial fluid promoting joint damage (Figure 2) (122–124).

### B Cells Have Emerged as Central Players in Systemic Lupus Erythematosus

Components of the innate and the adaptive immune system play an important role in the pathogenesis of systemic lupus erythematosus (SLE). Clinical manifestations of this autoimmune disease are diverse, affecting a wide spectrum of organs and tissues. The pathogenesis of the disease is not yet fully understood, but beside environmental factors a genetic susceptibility to SLE has been described including a variety of nucleotide polymorphisms [reviewed elsewhere (125)]. Plasmacytoid dendritic cells (pDC) produce type I interferon in response to viral infections. A large number of SLE patients possess an ongoing production of type I interferons and subsequently an increased expression of type I interferon regulated genes, termed IFN-signature, which correlates with autoantibodies and disease activity (126–128). This type I interferon synthesis is induced by immune complexes containing nucleic acid via Toll-like receptor (TLR) ligation. In addition to their antiviral features, type I interferons contribute to the activation of the adaptive immune system, e.g., by activation of autoreactive T and B cells (Figure 2) (129, 130). T cell signaling alterations and hyperactive B cells, producing and presenting autoantibodies against nuclear complexes to T cells, constitute the main drivers of SLE. The important role of B cells has been demonstrated in a murine model lacking this lymphocyte population (131). In addition to that, the same group showed that B cells also play an antibody-independent role in murine lupus in their function as antigen presenting and cytokine secreting cells (132).

Alterations in B cell maturation and differentiation affect several B cell subsets, targeting different checkpoints of B cell development. In SLE patients the frequency of antibody producing plasma cells in the peripheral blood is increased and correlates with autoantibody production and disease activity (133). It has been demonstrated that amongst others the overexpression of BAFF/BLyS (B-cell activating factor/B-lymphocyte stimulator), type I interferon and Blimp-1 (B lymphocyte-induced maturation protein-1) is responsible for these alterations in SLE patients (134–136).

Although B cells have emerged as central players in SLE, B cell depletion failed repeatedly as a therapeutic strategy in clinical trials. For example, the EXPLORER study demonstrated that rituximab, a CD20 antibody, did not show any statistically significant efficacy in achieving treatment response compared to placebo. Moreover, a recent reanalysis confirmed these findings, reevaluating the data with the help of newly available disease activity scores (137, 138).

There is only one therapeutic antibody approved by the FDA and the EMA for SLE therapy, namely belimumab, which neutralizes BAFF/BLyS and thereby decreases the number of newly formed B cells (139, 140).

The management of SLE strongly depends on the course of the disease. Glucocorticoids represent highly effective agents in order to immediately control the inflammatory process in SLE. Systemic glucocorticoids are required as initiation therapy in severe SLE, whereas maintenance immunosuppressive therapy is added in order to enable steroid tapering. Nevertheless, especially in acute, organ-threatening disease high-dose intravenous pulse therapy (usually 250–1,000 mg prednisone equivalent per day for 3 days) is often used to reduce disease activity (36). Interestingly, Guiducci et al. demonstrated that oral glucocorticoids (5–20 mg per day) modulate multiple gene expression pathways but the IFN pathway (including 36 type-I-IFN-inducible transcripts) is not affected in SLE patients. In contrast, the IFN signature was normalized after intravenous pulse therapy, which correlates with a reduction in pDC. The IFN-α production was reduced after a combined inhibition of TLR7 and 9 in purified pDC indicating that continuous triggering of TLR7 and 9 in these cells by immune complexes containing nucleic acid in SLE patients counteracts the activity of glucocorticoids on the IFN pathway. Thus, TLR7, and 9 inhibitors could be effective as glucocorticoid-sparing drugs (128).

However, the mechanism of glucocorticoid action in SLE patients is largely unknown. A study in MRL/MpSlac-lpr mice with systemic autoimmune symptoms similar to human SLE analyzed prednisone action on plasma cell differentiation with regard to the impact of regulatory factors, including IL-21, Blimp-1, and Bcl-6 (B cell lymphoma-6—essential for germinal center development). The percentages of plasma cells and plasma cell precursors as well as activated T cells were decreased after 13 weeks of prednisone treatment (Figure 2). In addition, serum IL-21 and the expression of splenic Blimp-1 and Bcl-6 were reduced, which may be correlated with the restriction of B lymphocyte differentiation into plasma cells in these mice (141).

Haneda et al. went further in order to analyze which step of B cell differentiation is affected by glucocorticoids. They differentiated human B cells by sequential addition of cytokines and other agents in a three-step culture system to obtain activated B cells [CD19(hi)CD38(lo)IgD(-)], plasmablasts [CD19(hi)CD38(hi)IgD(-)], and plasma cells [CD19(lo/-)CD38(hi)IgD(-)]. They added low and high concentrations of prednisolone at the beginning of each differentiation step and found a significant inhibition of B cell proliferation and differentiation in the last step, whereas IgG production was decreased in step 2 and 3 only at high glucocorticoid concentrations (100 ng/ml) (142). Interestingly, the number of circulating B cells was less affected by glucocorticoids compared to T cells which showed a rapid depletion in the circulation. In contrast, plasma cells and naive B cells are markedly decreased in the peripheral blood of SLE patients upon immunosuppressive therapy (143)—indicating that the inhibition of T cell help might contribute to the immediate glucocorticoid responses in SLE (144). Using transcriptome data to generate a pathway-level map of glucocorticoid effects across immune cell types, Franco et al. identified that glucocorticoid treatment (i) up-regulated the expression of *PRDM1*, which encodes BLIMP-1 involved in terminal differentiation and reduced proliferation of B cells and *IL10*, (ii) functionally impaired BCR signaling by suppressing *CR2* and *CD19* which encode the two components of the B cell co-receptor complex that serve as an enhancer of BCR-mediated signaling and (iii) selectively impaired TLR signaling by downregulation of *TLR1, TLR6*, and *TLR7* (32).

However, responses to glucocorticoids differ from patient to patient suffering from SLE. This may, at least in part, be related to the glucocorticoid receptor α whose alteration has been demonstrated in several autoimmune diseases (145–150). In SLE patients, the receptor expression is reduced compared to healthy controls. In addition, treatment with glucocorticoids further reduces the receptor mRNA and protein expression and it has been demonstrated that the receptor expression is negatively associated with SLE disease activity. Thus, the determination of receptor expression may be of importance with regard to insensitivity to glucocorticoids or determination of therapeutically effective dosages (151).

The glucocorticoid-induced leucine zipper (GILZ), an anti-inflammatory protein whose expression is upregulated by endogenous and exogenous glucocorticoids, has been in the focus of an *in vitro* study in human B cells. In general, GILZ mRNA and protein expression in peripheral blood mononuclear cells obtained from patients with SLE were downregulated compared to controls and correlated negatively with different markers of disease activity. An analysis of human B cell subsets revealed that intracellular GILZ was significantly decreased in circulating HLA-DR^lo^ plasmablasts [precursors of HLA-DR^hi^ cells which indicate active disease (152)] in patients with SLE. Treatment with prednisolone restored the GILZ expression to the level of control donors, a process described as transactivation—the activation of the transcription of anti-inflammatory proteins—in the introduction. Furthermore, an impaired induction of GILZ in SLE patients under glucocorticoid treatment was associated with an increased disease activity (153).

In the past decade, several additional factors including p-glycoprotein and the macrophage migration inhibitory factor (MIF) have been identified in the context of glucocorticoid resistance in SLE [reviewed in (154)]. P-glycoprotein (P-gp), a product of the multidrug resistance gene MDR-1, mediates the excretion of numerous drugs including antibiotics and cytotoxins but also glucocorticoids (155). P-gp is widely expressed in a variety of tissues, including peripheral blood T and B lymphocytes (156). However, P-gp expression is increased in these cells in SLE patients and is correlated with disease activity (157). Thus, elevated levels of P-gp lead to poor disease control by systemic glucocorticoid therapy and are associated with glucocorticoid resistance (157, 158). Beside P-gp, the inflammatory cytokine macrophage MIF actively reduces glucocorticoid action, participates in multiple stages of the inflammatory response and is widely associated with autoimmune disorders such as RA and SLE (159). MIF is also known as a naturally occurring counter-regulator of glucocorticoid action, correlates with disease activity in SLE and mediates the development of glucocorticoid resistance in SLE (159–162).

Although glucocorticoids are highly effective in the treatment of SLE, these drugs bear the risk of severe adverse effects, especially when given over a longer period of time and/or at higher dosages. A study analyzing the relationship between glucocorticoids and damage accrual in SLE demonstrated that medium to high mean daily prednisone doses and higher cumulative doses were associated with an increased occurrence of adverse effects. Eighteen patients developed new damage attributable to glucocorticoid treatment including cataracts, osteoporotic fractures, avascular necrosis, and diabetes mellitus (163). New drug developments or improved formulations for SLE therapy are promoted with the objective of reducing the glucocorticoid dosage and thereby attenuating adverse effects. The therapeutic effect of a liposome-based steroidal methylprednisolone nano-drug has been evaluated in a murine model of SLE compared to the free agent. The study revealed that the steroidal nano-drug formulation is significantly more effective in suppressing anti-dsDNA antibody levels, proliferation of lymphoid tissue and renal damage, and in prolonging survival compared to free methylprednisolone given at the same dosage (164). The advantage of nano-liposomes is that they passively reach the inflamed site due to the enhanced permeability of the inflamed tissue vasculature, ensuring a reduced level in non-inflamed tissues (165, 166). Due to these advantages, liposomal glucocorticoids are also of great interest in the treatment of other inflammatory diseases (167–170).

### Th2 Lymphocytes Constitute Major Contributors to the Pathogenesis of Allergic Diseases

Contrary to Th1 cells CD4+ T helper cells type 2 (Th2) are mainly involved in eosinophil activity as well as IgE production caused by an immunoglobulin class-switch in B cells (171). Th2 cells are characterized by the expression of GATA-3 and the secretion of Th2 cytokines, namely interleukin (IL)-4, IL-5, and IL-13 (172). Their development is promoted by a milieu abundant in IL-2 and IL-4 that activate STAT6 signaling and thereby promote Th2 differentiation (Figure 3). Thus, the key role of IL-4 consists in both mediating Th2 cell function and maintaining Th2 predominance by autocrine secretion.

Physiologically, Th2 cells exert their main function in the control of helminth infections. This mechanism of defense, referred to as the “type 2 response,” involves players of both the innate and adaptive immune system. Besides the activation and proliferation of Th2 cells and the secretion of their characteristic cytokines, this cascade comprises eosinophil and basophil granulocytes, mast cells as well as IgE secreted by plasma cells (173). Considered to possess anti-inflammatory characteristics, the type 2 response is thought to have evolved as a mechanism of parasite control that simultaneously confines collateral damage and promotes tissue repair (174). In this regard, the antibody isotype IgE fulfills an important function in responding to metazoan infections. Cross-linking of IgE bound to high-affinity receptors (FcεRI) on mast cells and basophils triggers the release of mediators that facilitate healing without activating complement. However, rising hygienic standards have reduced the necessity of antihelminthic defense mechanisms, thereby depriving Th2 cells of their original target pathogens. In this context, the role of a dysregulated type 2 response in the pathogenesis of immune-mediated diseases has attracted increasing attention.

With a lifetime prevalence of about 40%, allergic diseases represent the most common immune disorder in western countries, affecting both children and adults (175). The German Health Interview and Examination Survey for Children and Adolescents (KiGGS) revealed a prevalence of 22.6% among children and adolescents with three main diagnoses in descending order: atopic dermatitis (AD), allergic rhinoconjunctivitis (AR), and asthma (176). The pathogenesis of atopic disorders is defined by a predominant type 2 response involving all major players described above (177–180). The allergic cascade is set into motion by IL-4 and thymic stromal lymphopoietin (TSLP) secreted from basophils (181). This step promotes Th2 differentiation followed by the secretion of IL-4 and IL-13 from activated T lymphocytes (Figure 2). Subsequently, these cytokines cause B cells to undergo a class-switch to IgE producing plasma cells. Upon allergen exposure cross-linking of these antibodies bound to mast cells results in a release of histamine, prostaglandins, and leukotrienes that enhance paracellular permeability (Figure 2). As a result, dendritic cells (DCs) infiltrate the affected tissue and maintain T cell stimulation in their role as antigen-presenting cells (APCs). Activated Th2 cells produce type 2 cytokines that sustain the mechanisms underlying allergic reactions. While IL-4 mainly induces the class-switch toward IgE production, IL-13 additionally causes mucus production and airway hyperresponsiveness (182, 183). On the other hand, IL-5 supports eosinophil survival and function (Figure 2) (184, 185). The substantial role of the type 2 response in the pathogenesis of allergic diseases has also been highlighted by the examination of samples from patients suffering from AD (186), AR (187), and asthma (188–191) demonstrating the preponderance of Th2 cells and cytokines in the affected tissues.

Glucocorticoids, administered both topically and systemically, represent indispensable agents in the treatment of atopic disorders (192). Generally, these drugs are capable of reducing the number of immune cells present at the site of allergic reactions (Figure 2) (193, 194). On examining the effect of glucocorticoids on Th2 cells in greater detail, a contradiction becomes evident. Although these agents are successfully administered to atopic patients, glucocorticoids have been described to promote Th2 cell predominance (Figure 3)—a well-described driver of allergic diseases (195–201). In order to solve this apparent conflict, the mechanism of action of glucocorticoids in Th2-driven disorders needs to be reviewed more closely.

Firstly, one has to distinguish between short-term and long-term drug effects. Temporary application of supraphysiological glucocorticoid doses results in an inhibition of Th2 cytokine production (Figures 2, 3) (202–205). This effect is mainly mediated by glucocorticoid action on transcription factors as described in the introduction (206–208). For instance, binding of the GR to the IL-5 gene promoter region results in the repression of the cytokine by interfering with GATA-3 signaling (209). Moreover, this process seems to involve histone deacetylation. Similarly, inhibition of GATA-3, the key transcription factor of Th2 differentiation, plays an important role (210). Maneechotesuwan et al. showed that ligand-activated GR and GATA-3 compete for importin-alpha interaction enabling nuclear localization (211). Application of inhaled fluticasone propionate (FP) prevented nuclear transport of GATA-3 by means of this mechanism in asthmatic patients. The authors also demonstrated that the induction of MAPK phosphatase-1 (MPK-1) by FP results in the inhibition of p38 MAPK function, thus preventing GATA-3 phosphorylation. Also, dexamethasone treatment decreased GATA-3 expression in an asthmatic mouse model by inhibiting Notch1 signaling (212). In contrast, chronic exposure to glucocorticoids may cause a shift toward Th2 predominance. On the one hand, this thesis is underlined by multiple studies analyzing the role of stress in atopic diseases. Periods of stress are marked by elevated levels of endogenous cortisol that promote Th2 predominance and thereby susceptibility to allergy (213). The impact of psychological stress on the course of disease in asthmatic patients has been reviewed by Miyasaka et al. (214). On the other hand, Ramirez revealed that prior glucocorticoid exposure provokes type 2 cytokine production in T cells (201).

Secondly, the effect of glucocorticoid administration on Th2 cells in atopic patients appears to differ from the Th2 enhancement generally caused by these drugs. Hydrocortisone significantly reduced the presence of IgE, histamine, and type 2 cytokines in serum and skin samples from AD patients (215). Correspondingly, AR patients presented a decrease in eosinophils, IgE, IL-4, and IL-5 in their nasal fluid after topical and oral glucocorticoid application (Figure 2) (187, 216–218). Lastly, the suppression of the type 2 response by glucocorticoid treatment was also observed in bronchial tissue and bronchoalveolar lavage fluid from asthmatics (219–221).

Finally, the beneficial glucocorticoid actions in allergic diseases are not only caused by their impact on Th2 lymphocytes. On the contrary, several players of the type 2 immune response are equally affected by glucocorticoid treatment. Namely, mast cell maturation and activation, FcεRI expression as well as mediator production and release are inhibited by glucocorticoid exposure (222–227). Furthermore, glucocorticoids impede histamine release from basophils and induce eosinophil apoptosis (228, 229). Recruitment and function of APCs as well as the class-switch to IgE in B cells are also restrained (230–232). Additionally, Klaßen et al. demonstrated the importance of non-hematopoietic cells in mediating glucocorticoid effects in a mouse model of allergic asthma (233). In the end, it has to be mentioned that recent findings emphasize the involvement of other CD4+ T helper cell subsets in the pathogenesis of allergic diseases. Increasing importance has been ascribed to Th17, Th9, Th22, and Th25 cells in this context (234). Similarly, the impact of regulatory T cells (Treg) must not be neglected. Several studies describe defective Treg activity as a major contributor to the development and maintenance of atopy (235–242). In this regard, glucocorticoids greatly contribute to the restoration of Treg function, thereby controlling the dysregulated type 2 immune response (243–246).

## Mechanisms of Glucocorticoids in the Treatment of Malignancies With a Focus on Leukemia

The last chapter will give a brief insight into the mechanisms of glucocorticoid action in cancer therapies. Interestingly, the effects of glucocorticoids on different cancer subtypes and thereby the underlying mechanisms vary, even regarding opposite effects. This may be related to the subtype of cancer itself including its location, the affected cell type, the microenvironment and emerging comorbidities. Also, the glucocorticoid dose ranging from low to high daily dosages, and the level of glucocorticoid receptor expression and activity play an important role. In addition to that, the co-existence of other receptors of the steroid receptor family, namely the androgen and the estrogen receptors, can affect glucocorticoid action, especially in breast or prostate cancer, since there are also differences in receptor positive and receptor negative cancer subtypes. Another beneficial effect on different subtypes of cancer should not be neglected: Glucocorticoids are used as co-therapy during chemotherapy or radiotherapy in order to reduce side effects. They have been shown to improve mood, increase appetite and thereby lessen weight loss, reduce fatigue, diminish ureteric obstruction, prevent vomiting, and alleviate pain (247–250).

In the following, we will concentrate on hematopoietic malignancies which form a particular subset of cancerous conditions that were first discovered as such in the Nineteenth century when Rudolf Virchow coined the term “leukemia,” meaning “white blood.” Glucocorticoids play a crucial role in the treatment of these malignancies, among others as part of the CHOP regimen to treat non-Hodgkin lymphoma as well as in myeloma therapy. Nevertheless, due to the considerable differences of glucocorticoid effects on diverse cancer types, we will focus on one subtype here, namely leukemia. The epidemiology of the disease is summarized in Table 2 according to the German and Austrian cancer register (www.gekid.de; www.statistik.at).

**Table 2:** Epidemiology of leukemia.

**Registry**	**Cancer type**	**Female**		**Male**		**Total**
		**Rate**	**Number of cases**	**Rate**	**Number of cases**	**Number of cases**
Germany	Cancer (total)	336.7	223.019	436.5	259.013	482.032
	Leukemia	8.1	5.550	13.2	7.489	13.039
	Leukemia (mortality)	4.0	3.575	6.4	4.168	7.743
Austria	Cancer (total)	421.8	19.393	581.4	21.342	40.735
	Leukemia	9.0	420	16.5	586	1.006
	Leukemia (mortality)	7.3	354	11.9	386	740

*Rate and absolute numbers of cases of total cancer (without other tumors of the skin) and leukemic (including mortality) in Germany and Austria in the year 2014. Rate is given per 100,000 individuals per year (new disease) according to the German and Austrian cancer registry (www.gekid.de; www.statistik.at)*.

Four different types of leukemia are described: chronic lymphoblastic leukemia (CLL), chronic myeloid leukemia (CML), acute myeloid leukemia (AML), and acute lymphoblastic leukemia (ALL). CLL is the most common leukemic disease in western industrialized countries, where the disease constitutes 95% of the overall cases in older individuals (50 years and older) (251). The main reason for the therapeutic use of glucocorticoids in leukemia is their pro-apoptotic action.

### Chronic Lymphoblastic Leukemia (CLL)

The inhibition of B cell apoptosis and the dysregulation of proliferation and differentiation are the main causes of CLL. They lead to an accumulation of mature CD5-positive, CD10-negative, CD20 weakly positive, and CD23-positive B cells within blood, bone marrow and solid lymphoid organs (252–254). Therefore, the B cell itself, the B cell receptor and the subsequent signaling pathways are novel targets of therapies using e.g., monoclonal antibodies like rituximab or small molecules such as the kinase inhibitor ibrutinib [reviewed in (253)].

In healthy subjects, the administration of glucocorticoids affects different subsets of the peripheral blood leukocytes, resulting in a transient lymphocytopenia (255). This has been demonstrated to be mostly caused by a glucocorticoid induced redistribution of lymphocytes from the blood into the tissue, affecting mainly T cells and B cells to a lesser extent (252, 256). In CLL patients however, the administration of glucocorticoids leads to an increase in blood lymphocytes accompanied by a rapid reduction in spleen and lymph node sizes. Following the therapy, the number of lymphocytes decreased even beneath pretreatment levels (257, 258). Unfortunately, the underlying mechanism is still unknown and glucocorticoids are consequently not commonly used to treat CLL. Nevertheless, these drugs are currently of interest to complement treatment with monoclonal antibodies or small molecules. In 2016, Manzoni et al. analyzed the *in vitro* effects of the combination of ibrutinib and dexamethasone on the proliferation and metabolic stress markers in lymphocytes obtained from patients suffering from CLL. They demonstrated an enhanced inhibition of cell cycle progression, an increase in apoptosis and a decrease in DNA damage in lymphoid B cells by a combination of dexamethasone and ibrutinib compared to the tyrosine kinase inhibitor alone (259).

### Chronic Myeloid Leukemia (CML)

Tyrosine kinase inhibitors also show remarkable success in controlling CML, a disease of myeloid progenitor cells. This is due to the knowledge of the underlying molecular pathogenesis of this disease which arises mainly from a translocation *t*_(9, 22)_ (q34;q11), resulting in transcripts and fusion proteins with unusual tyrosine kinase activity (260). Thus, tyrosine kinase inhibitors, e.g., imatinib and dasatinib, are used as standard therapy with a high rate of remission (261). Consequently, the use of glucocorticoids has become dispensable. Unfortunately, this kind of molecular-targeted therapy is exceptional since the molecular target is unknown in all other types of leukemia.

### Acute Myeloid Leukemia (AML)

The heterogeneous character of AML impedes such targeted therapies. Therefore, the treatment largely relies on the use of aggressive chemotherapy (262). AML is characterized by an infiltration of the bone marrow, blood, and tissues by hematopoietic progenitor cells which lose their ability to differentiate physiologically due to heterogeneous clonal disorders. The extent of the genetic variability of AML patients has been the focus of different studies aiming at customized therapeutic approaches (263). In contrast to more recent findings, it has been demonstrated in 2006 that short-term treatment with high-dose methylprednisolone resulted in an induction of differentiation and apoptosis of leukemic cells in children with AML. Furthermore, the addition of this high-dose glucocorticoid therapy to chemotherapy led to increased remission rates and improved patient outcome (264). However, high rates of glucocorticoid resistance in AML patients have been reported in the last years, so that glucocorticoids are not suitable as standard therapy (265).

### Acute Lymphoblastic Leukemia (ALL)

In contrast, leukemic cells in ALL are much more sensitive to glucocorticoids. Therefore, the administration of high-dose glucocorticoids (i.e., dexamethasone and prednisolone) represents the standard induction therapy in ALL (266). The specific genotypes of ALL are diverse, including aberrant expression of proto-oncogenes, chromosomal translocations resulting in fusion genes and hyperdiploidy involving more than 50 chromosomes [reviewed in (267)]. These genetic alterations contribute to changes in cellular function, such as a dysregulation of differentiation, proliferation, and programmed cell death of hematopoietic stem cells (254, 267, 268).

The glucocorticoid-induced cell death in leukemia is mediated by the glucocorticoid receptor via transrepression and transactivation (please see Introduction). It has been demonstrated that the repression of anti-apoptotic BCL2 and the activation of the antagonizing pro-apoptotic BIM induce cell death in ALL (269, 270). Other genes and even microRNAs have been described to be regulated by glucocorticoids and thereby mediate apoptosis (271–273). In addition, cell death is also triggered by calcium release from the endoplasmic reticulum into the cytosol and by an enhanced expression of thioredoxin-interacting protein (TXNIP) which induces cell death by increasing reactive oxygen species and/or blocking glucose transport (270).

Finally, the underlying mechanisms which mediate glucocorticoid-induced cell death in leukemia are diverse and not yet well-understood. The existence and the development of glucocorticoid resistance after long-term therapy aggravate treatment strategies or reverse the achieved remission. This applies to both the treatment of cancer and the treatment of inflammatory autoimmune diseases.

## Concluding Remarks

After highlighting the effects of glucocorticoids in different immune cells in the context of a variety of immunopathologies, we have to conclude that the understanding of the mode of glucocorticoid action in the scope of immune responses and glucocorticoid resistance is still incomplete. Although glucocorticoids have ranked among the most potent immunosuppressive drugs in daily clinical care for more than 70 years, knowledge on their mechanisms of action on cellular and sub-cellular levels in an immune cell type-specific manner and in the context of the respective immunopathology remains scarce. Further research into this topic will enhance our comprehension of the capacity spectrum of glucocorticoid action and the establishment of glucocorticoid resistance, also providing guidance for personalized therapy.

## Author Contributions

TG, LE, and CS designed the concept. CS and LE prepared the figures. All authors wrote the manuscript and read and approved the final version of the manuscript.

### Conflict of Interest Statement

FB reports receiving consultancy fees, honoraria and/or travel expenses, and grant/study support from Horizon Pharma and Mundipharma. The remaining authors declare that the research was conducted in the absence of any commercial or financial relationships that could be construed as a potential conflict of interest.

## References

[1] Buttgereit F da Silva JA Boers M Burmester GR Cutolo M Jacobs J. Standardised nomenclature for glucocorticoid dosages and glucocorticoid treatment regimens: current questions and tentative answers in rheumatology. Ann Rheum Dis. (2002) 61:718–22. 10.1136/ard.61.8.718 12117678 PMC1754188

[2] Miller WL. Steroidogenesis: unanswered questions. Trends Endocrinol. Metab. (2017) 28:771–93. 10.1016/j.tem.2017.09.002 29031608

[3] Buttgereit F Zhou H Seibel MJ. Arthritis and endogenous glucocorticoids: the emerging role of the 11β-HSD enzymes. Ann Rheum Dis. (2008) 67:1201. 10.1136/ard.2008.092502 18697779

[4] Munck A Naray-Fejes-Toth A. Glucocorticoids and stress: permissive and suppressive actions. Ann N Y Acad Sci. (1994) 746:115–30. Discussion 31–3. 10.1111/j.1749-6632.1994.tb39221.x 7825870

[5] Almawi WY Melemedjian OK. Molecular mechanisms of glucocorticoid antiproliferative effects: antagonism of transcription factor activity by glucocorticoid receptor. J Leukoc Biol. (2002) 71:9–15. 10.1189/jlb.71.1.9 11781376

[6] Hutchison KA Scherrer LC Czar MJ Ning Y Sanchez ER Leach KL FK506 binding to the 56-kilodalton immunophilin (Hsp56) in the glucocorticoid receptor heterocomplex has no effect on receptor folding or function. Biochemistry. (1993) 32:3953–7. 10.1021/bi00066a015 7682438

[7] Pratt WB. The hsp90-based chaperone system: involvement in signal transduction from a variety of hormone and growth factor receptors. Proc Soc Exp Biol Med. (1998) 217:420–34. 10.3181/00379727-217-44252 9521088

[8] Pratt W Morishima Y Murphy M Harrell M Chaperoning of glucocorticoid receptors. In: Starke K Gaestel M, editors. Molecular Chaperones in Health and Disease. Handbook of Experimental Pharmacology, vol. 172 Berlin; Heidelberg: Springer (2006). 10.1007/3-540-29717-0_5 16610357

[9] Wikstrom AC. Glucocorticoid action and novel mechanisms of steroid resistance: role of glucocorticoid receptor-interacting proteins for glucocorticoid responsiveness. J Endocrinol. (2003) 178:331–7. 10.1677/joe.0.1780331 12967326

[10] Hadji P Kyvernitakis I Kann PH Niedhart C Hofbauer LC Schwarz H GRAND-4: the German retrospective analysis of long-term persistence in women with osteoporosis treated with bisphosphonates or denosumab. Osteoporosis Int. (2016) 27:2967–78. 10.1007/s00198-016-3623-6 PMC5042990 27172934

[11] Hollenberg SM Evans RM. Multiple and cooperative trans-activation domains of the human glucocorticoid receptor. Cell. (1988) 55:899–906. 10.1016/0092-8674(88)90145-6 3191531

[12] Oakley RH Cidlowski JA. Cellular processing of the glucocorticoid receptor gene and protein: new mechanisms for generating tissue specific actions of glucocorticoids. J Biol Chem. (2010) 286:3177–84. 10.1074/jbc.R110.179325 21149445 PMC3030321

[13] Oakley RH Sar M Cidlowski JA. The human glucocorticoid receptor beta isoform. Expression, biochemical properties, and putative function. J Biol Chem. (1996) 271:9550–9. 10.1074/jbc.271.16.9550 8621628

[14] Ismaili N Garabedian MJ. Modulation of glucocorticoid receptor function via phosphorylation. Ann N Y Acad Sci. (2004) 1024:86–101. 10.1196/annals.1321.007 15265775

[15] Stahn C Buttgereit F. Genomic and nongenomic effects of glucocorticoids. Nat Clin Pract Rheumatol. (2008) 4:525–33. 10.1038/ncprheum0898 18762788

[16] Stahn C Lowenberg M Hommes DW Buttgereit F. Molecular mechanisms of glucocorticoid action and selective glucocorticoid receptor agonists. Mol Cell Endocrinol. (2007) 275:71–8. 10.1016/j.mce.2007.05.019 17630118

[17] Mercieca C Kirwan JR. The intelligent use of systemic glucocorticoids in rheumatoid arthritis. Expert Rev Clin Immunol. (2014) 10:143–57. 10.1586/1744666X.2014.864236 24308837

[18] Strehl C Bijlsma JW de Wit M Boers M Caeyers N Cutolo M. Defining conditions where long-term glucocorticoid treatment has an acceptably low level of harm to facilitate implementation of existing recommendations: viewpoints from an EULAR task force. Ann Rheum Dis. (2016) 75:952–7. 10.1136/annrheumdis-2015-208916 26933146

[19] Belvisi MG Wicks SL Battram CH Bottoms SE Redford JE Woodman P. Therapeutic benefit of a dissociated glucocorticoid and the relevance of *in vitro* separation of transrepression from transactivation activity. J Immunol. (2001) 166:1975–82. 10.4049/jimmunol.166.3.1975 11160246

[20] Schacke H Schottelius A Docke WD Strehlke P Jaroch S Schmees N. Dissociation of transactivation from transrepression by a selective glucocorticoid receptor agonist leads to separation of therapeutic effects from side effects. Proc Natl Acad Sci USA. (2004) 101:227–32. 10.1073/pnas.0300372101 14694204 PMC314167

[21] Song RX Barnes CJ Zhang Z Bao Y Kumar R Santen RJ. The role of Shc and insulin-like growth factor 1 receptor in mediating the translocation of estrogen receptor alpha to the plasma membrane. Proc Natl Acad Sci USA. (2004) 101:2076–81. 10.1073/pnas.0308334100 14764897 PMC357054

[22] Vandevyver S Dejager L Tuckermann J Libert C. New insights into the anti-inflammatory mechanisms of glucocorticoids: an emerging role for glucocorticoid-receptor-mediated transactivation. Endocrinology. (2013) 154:993–1007. 10.1210/en.2012-2045 23384835

[23] Ratman D Vanden Berghe W Dejager L Libert C Tavernier J Beck IM. How glucocorticoid receptors modulate the activity of other transcription factors: a scope beyond tethering. Mol Cell Endocrinol. (2013) 380:41–54. 10.1016/j.mce.2012.12.014 23267834

[24] Reichardt HM Tuckermann JP Gottlicher M Vujic M Weih F Angel P. Repression of inflammatory responses in the absence of DNA binding by the glucocorticoid receptor. EMBO J. (2001) 20:7168–73. 10.1093/emboj/20.24.7168 11742993 PMC125338

[25] Kleiman A Tuckermann JP. Glucocorticoid receptor action in beneficial and side effects of steroid therapy: lessons from conditional knockout mice. Mol Cell Endocrinol. (2007) 275:98–108. 10.1016/j.mce.2007.05.009 17587493

[26] Tuckermann JP Kleiman A Moriggl R Spanbroek R Neumann A Illing A. Macrophages and neutrophils are the targets for immune suppression by glucocorticoids in contact allergy. J Clin Invest. (2007) 117:1381–90. 10.1172/JCI28034 17446934 PMC1849982

[27] McDonough AK Curtis JR Saag KG. The epidemiology of glucocorticoid-associated adverse events. Curr Opin Rheumatol. (2008) 20:131–7. 10.1097/BOR.0b013e3282f51031 18349741

[28] Schacke H Docke WD Asadullah K. Mechanisms involved in the side effects of glucocorticoids. Pharmacol Ther. (2002) 96:23–43. 10.1016/S0163-7258(02)00297-8 12441176

[29] Murphy K Travers P Walport M Janeway C Janeway's Immunobiology. 8th ed New York, NY: Garland Science (2012). p. 275–315.

[30] Nicholson LB. The immune system. Essays Biochem. (2016) 60:275–301. 10.1042/EBC20160017 27784777 PMC5091071

[31] Oppong E Cato AC. Effects of glucocorticoids in the immune system. Adv Exp Med Biol. (2015) 872:217–33. 10.1007/978-1-4939-2895-8_9 26215996

[32] Franco LM Gadkari M Howe KN Sun J Kardava L Kumar P. Immune regulation by glucocorticoids can be linked to cell type–dependent transcriptional responses. J Exp Med. (2019) 216:384–406. 10.1084/jem.20180595 30674564 PMC6363437

[33] Hellmich B Agueda A Monti S Buttgereit F de Boysson H Brouwer E. 2018 Update of the EULAR recommendations for the management of large vessel vasculitis. Ann Rheum Dis. (2019). 10.1136/annrheumdis-2019-215672. [Epub ahead of print]. 31270110

[34] Buttgereit F Dejaco C Matteson EL Dasgupta B. Polymyalgia rheumatica and giant cell arteritis: a systematic review. JAMA. (2016) 315:2442–58. 10.1001/jama.2016.5444 27299619

[35] Smolen JS Landewé R Bijlsma J Burmester G Chatzidionysiou K Dougados M. EULAR recommendations for the management of rheumatoid arthritis with synthetic and biological disease-modifying antirheumatic drugs: 2016 update. Ann Rheum Dis. (2017) 76:960–77. 10.1136/annrheumdis-2016-210715 28264816

[36] Fanouriakis A Kostopoulou M Alunno A Aringer M Bajema I Boletis JN. 2019 update of the EULAR recommendations for the management of systemic lupus erythematosus. Ann Rheum Dis. (2019) 78:736–45. 10.1136/annrheumdis-2019-215089 30926722

[37] Wollenberg A Barbarot S Bieber T Christen-Zaech S Deleuran M Fink-Wagner A Consensus-based European guidelines for treatment of atopic eczema (atopic dermatitis) in adults and children: part I. J Eur Acad Dermatol Venereol. (2018) 32:657–82. 10.1111/jdv.14891 29676534

[38] Wuthrich B Schmid-Grendelmeier P. The atopic eczema/dermatitis syndrome. Epidemiology, natural course, and immunology of the IgE-associated (“extrinsic”) and the nonallergic (“intrinsic”) AEDS. J Investig Allergol Clin Immunol. (2003) 13:1–5. 10.3109/9780203908877-2 12861844

[39] Wollenberg A Oranje A Deleuran M Simon D Szalai Z Kunz B. ETFAD/EADV Eczema task force 2015 position paper on diagnosis and treatment of atopic dermatitis in adult and paediatric patients. J Eur Acad Dermatol Venereol. (2016) 30:729–47. 10.1111/jdv.13599 27004560

[40] Wheatley LM Togias A. Clinical practice. Allergic rhinitis. N Engl J Med. (2015) 372:456–63. 10.1056/NEJMcp1412282 25629743 PMC4324099

[41] Greiner AN Hellings PW Rotiroti G Scadding GK. Allergic rhinitis. Lancet. (2011) 378:2112–22. 10.1016/S0140-6736(11)60130-X 21783242

[42] Brozek JL Bousquet J Agache I Agarwal A Bachert C Bosnic-Anticevich S. Allergic Rhinitis and its Impact on Asthma (ARIA) guidelines-2016 revision. J Allergy Clin Immunol. (2017) 140:950–8. 10.1016/j.jaci.2017.03.050 28602936

[43] O'Byrne PM FitzGerald JM Bateman ED Barnes PJ Zhong N Keen C. Inhaled combined budesonide-formoterol as needed in mild asthma. N Engl J Med. (2018) 378:1865–76. 10.1056/NEJMoa1715274 29768149

[44] O'Byrne PM Naya IP Kallen A Postma DS Barnes PJ. Increasing doses of inhaled corticosteroids compared to adding long-acting inhaled beta2-agonists in achieving asthma control. Chest. (2008) 134:1192–9. 10.1378/chest.08-1018 18689590

[45] Chung KF Wenzel SE Brozek JL Bush A Castro M Sterk PJ. International ERS/ATS guidelines on definition, evaluation and treatment of severe asthma. Eur Respir J. (2014) 43:343–73. 10.1183/09031936.00202013 24337046

[46] Rank MA Hagan JB Park MA Podjasek JC Samant SA Volcheck GW. The risk of asthma exacerbation after stopping low-dose inhaled corticosteroids: a systematic review and meta-analysis of randomized controlled trials. J Allergy Clin Immunol. (2013) 131:724–9. 10.1016/j.jaci.2012.11.038 23321206

[47] Hagan JB Samant SA Volcheck GW Li JT Hagan CR Erwin PJ. The risk of asthma exacerbation after reducing inhaled corticosteroids: a systematic review and meta-analysis of randomized controlled trials. Allergy. (2014) 69:510–6. 10.1111/all.12368 24571355

[48] Thomas A Lemanske RF Jr Jackson DJ. Approaches to stepping up and stepping down care in asthmatic patients. J Allergy Clin Immunol. (2011) 128:915–24. 10.1016/j.jaci.2011.07.014 21855125 PMC3205296

[49] Hasegawa T Ishihara K Takakura S Fujii H Nishimura T Okazaki M. Duration of systemic corticosteroids in the treatment of asthma exacerbation; a randomized study. Intern Med. (2000) 39:794–7. 10.2169/internalmedicine.39.794 11030202

[50] Jones AM Munavvar M Vail A Aldridge RE Hopkinson L Rayner C. Prospective, placebo-controlled trial of 5 vs 10 days of oral prednisolone in acute adult asthma. Respir Med. (2002) 96:950–4. 10.1053/rmed.2002.1369 12418594

[51] Choo KJ Simons E Sheikh A. Glucocorticoids for the treatment of anaphylaxis: cochrane systematic review. Allergy. (2010) 65:1205–11. 10.1111/j.1398-9995.2010.02424.x 20584003

[52] Muraro A Werfel T Hoffmann-Sommergruber K Roberts G Beyer K Bindslev-Jensen C. EAACI food allergy and anaphylaxis guidelines: diagnosis and management of food allergy. Allergy. (2014) 69:1008–25. 10.1111/all.12429 24909706

[53] Ring J Beyer K Biedermann T Bircher A Duda D Fischer J. Guideline for acute therapy and management of anaphylaxis: S2 Guideline of the German Society for Allergology and Clinical Immunology (DGAKI), the Association of German Allergologists (AeDA), the Society of Pediatric Allergy and Environmental Medicine (GPA), the German Academy of Allergology and Environmental Medicine (DAAU), the German Professional Association of Pediatricians (BVKJ), the Austrian Society for Allergology and Immunology (OGAI), the Swiss Society for Allergy and Immunology (SGAI), the German Society of Anaesthesiology and Intensive Care Medicine (DGAI), the German Society of Pharmacology (DGP), the German Society for Psychosomatic Medicine (DGPM), the German Working Group of Anaphylaxis Training and Education (AGATE) and the patient organization German Allergy and Asthma Association (DAAB). Allergo J Int. (2014) 23:96–112. 10.1007/s40629-014-0009-1 26120521 PMC4479483

[54] Eichhorst B Robak T Montserrat E Ghia P Hillmen P Hallek M. Chronic lymphocytic leukaemia: ESMO Clinical Practice Guidelines for diagnosis, treatment and follow-up. Ann Oncol. (2015) 26(Suppl. 5):v78–84. 10.1093/annonc/mdv303 26314781

[55] Willemze R Hodak E Zinzani PL Specht L Ladetto M Committee EG. Primary cutaneous lymphomas: ESMO Clinical Practice Guidelines for diagnosis, treatment and follow-up. Ann Oncol. (2018) 29(Supplement_4):iv30–40. 10.1093/annonc/mdy133 29878045

[56] Eichenauer DA Aleman BMP Andre M Federico M Hutchings M Illidge T. Hodgkin lymphoma: ESMO Clinical Practice Guidelines for diagnosis, treatment and follow-up. Ann Oncol. (2018) 29(Supplement_4):iv19–29. 10.1093/annonc/mdy080 29796651

[57] Hoelzer D Bassan R Dombret H Fielding A Ribera JM Buske C. Acute lymphoblastic leukaemia in adult patients: ESMO Clinical Practice Guidelines for diagnosis, treatment and follow-up. Ann Oncol. (2016) 27(suppl. 5):v69–82. 10.1093/annonc/mdw025 27056999

[58] Jennette JC Falk RJ Bacon PA Basu N Cid MC Ferrario F. 2012 revised International Chapel Hill Consensus Conference Nomenclature of Vasculitides. Arthritis Rheum. (2013) 65:1–11. 10.1002/art.37715 23045170

[59] Terrades-Garcia N Cid MC. Pathogenesis of giant-cell arteritis: how targeted therapies are influencing our understanding of the mechanisms involved. Rheumatology. (2018) 57(suppl_2):ii51–i62. 10.1093/rheumatology/kex423 29982777

[60] Al-Mousawi AZ Gurney SP Lorenzi AR Pohl U Dayan M Mollan SP. Reviewing the pathophysiology behind the advances in the management of giant cell arteritis. Ophthalmol Ther. (2019) 8:177–93. 10.1007/s40123-019-0171-0 30820767 PMC6513947

[61] Hernandez-Rodriguez J Segarra M Vilardell C Sanchez M Garcia-Martinez A Esteban MJ. Tissue production of pro-inflammatory cytokines (IL-1beta, TNFalpha and IL-6) correlates with the intensity of the systemic inflammatory response and with corticosteroid requirements in giant-cell arteritis. Rheumatology. (2004) 43:294–301. 10.1093/rheumatology/keh058 14679293

[62] Watanabe R Maeda T Zhang H Berry GJ Zeisbrich M Brockett R. MMP (Matrix Metalloprotease)-9-producing monocytes enable T cells to invade the vessel wall and cause vasculitis. Circ Res. (2018) 123:700–15. 10.1161/CIRCRESAHA.118.313206 29970365 PMC6202245

[63] Stone JH Tuckwell K Dimonaco S Klearman M Aringer M Blockmans D Trial of tocilizumab in giant-cell arteritis. N Engl J Med. (2017) 377:317–28. 10.1056/NEJMoa1613849 28745999

[64] Matteson EL Buttgereit F Dejaco C Dasgupta B. Glucocorticoids for management of polymyalgia rheumatica and giant cell arteritis. Rheum Dis Clin North Am. (2016) 42:75–90. 10.1016/j.rdc.2015.08.009 26611552

[65] Brack A Rittner HL Younge BR Kaltschmidt C Weyand CM Goronzy JJ. Glucocorticoid-mediated repression of cytokine gene transcription in human arteritis-SCID chimeras. J Clin Invest. (1997) 99:2842–50. 10.1172/JCI119477 9185506 PMC508134

[66] Weyand CM Kaiser M Yang H Younge B Goronzy JJ. Therapeutic effects of acetylsalicylic acid in giant cell arteritis. Arthritis Rheum. (2002) 46:457–66. 10.1002/art.10071 11840449

[67] Yamamoto Y Gaynor RB. Therapeutic potential of inhibition of the NF-kappaB pathway in the treatment of inflammation and cancer. J Clin Invest. (2001) 107:135–42. 10.1172/JCI11914 11160126 PMC199180

[68] Linden M Brattsand R. Effects of a corticosteroid, budesonide, on alveolar macrophage and blood monocyte secretion of cytokines: differential sensitivity of GM-CSF, IL-1 beta, and IL-6. Pulm Pharmacol. (1994) 7:43–7. 10.1006/pulp.1994.1004 8003851

[69] Cronstein BN Kimmel SC Levin RI Martiniuk F Weissmann G. A mechanism for the antiinflammatory effects of corticosteroids: the glucocorticoid receptor regulates leukocyte adhesion to endothelial cells and expression of endothelial-leukocyte adhesion molecule 1 and intercellular adhesion molecule 1. Proc Natl Acad Sci USA. (1992) 89:9991–5. 10.1073/pnas.89.21.9991 1279685 PMC50263

[70] Blotta MH DeKruyff RH Umetsu DT. Corticosteroids inhibit IL-12 production in human monocytes and enhance their capacity to induce IL-4 synthesis in CD4+ lymphocytes. J Immunol. (1997) 158:5589–95. 9190905

[71] Deng J Younge BR Olshen RA Goronzy JJ Weyand CM. Th17 and Th1 T-cell responses in giant cell arteritis. Circulation. (2010) 121:906–15. 10.1161/CIRCULATIONAHA.109.872903 20142449 PMC2837465

[72] Espigol-Frigole G Corbera-Bellalta M Planas-Rigol E Lozano E Segarra M Garcia-Martinez A. Increased IL-17A expression in temporal artery lesions is a predictor of sustained response to glucocorticoid treatment in patients with giant-cell arteritis. Ann Rheum Dis. (2013) 72:1481–7. 10.1136/annrheumdis-2012-201836 22993227

[73] Weyand CM Goronzy JJ. Immune mechanisms in medium and large-vessel vasculitis. Nat Rev Rheumatol. (2013) 9:731–40. 10.1038/nrrheum.2013.161 24189842 PMC4277683

[74] Saadoun D Garrido M Comarmond C Desbois AC Domont F Savey L. Th1 and Th17 cytokines drive inflammation in Takayasu arteritis. Arthritis Rheumatol. (2015) 67:1353–60. 10.1002/art.39037 25604824

[75] Samson M Audia S Fraszczak J Trad M Ornetti P Lakomy D. Th1 and Th17 lymphocytes expressing CD161 are implicated in giant cell arteritis and polymyalgia rheumatica pathogenesis. Arthritis Rheum. (2012) 64:3788–98. 10.1002/art.34647 22833233

[76] Corbera-Bellalta M Garcia-Martinez A Lozano E Planas-Rigol E Tavera-Bahillo I Alba MA. Changes in biomarkers after therapeutic intervention in temporal arteries cultured in Matrigel: a new model for preclinical studies in giant-cell arteritis. Ann Rheum Dis. (2014) 73:616–23. 10.1136/annrheumdis-2012-202883 23625984

[77] Samson M Corbera-Bellalta M Audia S Planas-Rigol E Martin L Cid MC. Recent advances in our understanding of giant cell arteritis pathogenesis. Autoimmun Rev. (2017) 16:833–44. 10.1016/j.autrev.2017.05.014 28564617

[78] Smolen JS Aletaha D Barton A Burmester GR Emery P Firestein GS Rheumatoid arthritis. Nat Rev Dis Primers. (2018) 4:18001 10.1038/nrdp.2018.2 29417936

[79] Ospelt C. Synovial fibroblasts in 2017. RMD Open. (2017) 3:e000471. 10.1136/rmdopen-2017-000471 29081987 PMC5652455

[80] Neeck G. Fifty years of experience with cortisone therapy in the study and treatment of rheumatoid arthritis. Ann N Y Acad Sci. (2002) 966:28–38. 10.1111/j.1749-6632.2002.tb04199.x 12114256

[81] Kirwan J Power L. Glucocorticoids: action and new therapeutic insights in rheumatoid arthritis. Curr Opin Rheumatol. (2007) 19:233–7. 10.1097/BOR.0b013e3280d6471a 17414948

[82] Strehl C van der Goes MC Bijlsma JW Jacobs JW Buttgereit F. Glucocorticoid-targeted therapies for the treatment of rheumatoid arthritis. Expert Opin Invest Drugs. (2017) 26:187–95. 10.1080/13543784.2017.1276562 28043173

[83] Capell HA Madhok R Hunter JA Porter D Morrison E Larkin J. Lack of radiological and clinical benefit over two years of low dose prednisolone for rheumatoid arthritis: results of a randomised controlled trial. Ann Rheum Dis. (2004) 63:797–803. 10.1136/ard.2003.014050 15194574 PMC1755058

[84] Wassenberg S Rau R Steinfeld P Zeidler H. Very low-dose prednisolone in early rheumatoid arthritis retards radiographic progression over two years: a multicenter, double-blind, placebo-controlled trial. Arthritis Rheum. (2005) 52:3371–80. 10.1002/art.21421 16255011

[85] Quinn MA Green MJ Marzo-Ortega H Proudman S Karim Z Wakefield RJ. Prognostic factors in a large cohort of patients with early undifferentiated inflammatory arthritis after application of a structured management protocol. Arthritis Rheum. (2003) 48:3039–45. 10.1002/art.11269 14613264

[86] Machold KP Landewe R Smolen JS Stamm TA van der Heijde DM Verpoort KN. The Stop Arthritis Very Early (SAVE) trial, an international multicentre, randomised, double-blind, placebo-controlled trial on glucocorticoids in very early arthritis. Ann Rheum Dis. (2010) 69:495–502. 10.1136/ard.2009.122473 20223838

[87] Verstappen SM McCoy MJ Roberts C Dale NE Hassell AB Symmons DP. Beneficial effects of a 3-week course of intramuscular glucocorticoid injections in patients with very early inflammatory polyarthritis: results of the STIVEA trial. Ann Rheum Dis. (2010) 69:503–9. 10.1136/ard.2009.119149 19825849

[88] Damsker JM Hansen AM Caspi RR. Th1 and Th17 cells: adversaries and collaborators. Ann N Y Acad Sci. (2010) 1183:211–21. 10.1111/j.1749-6632.2009.05133.x 20146717 PMC2914500

[89] Thomas R McIlraith M Davis LS Lipsky PE. Rheumatoid synovium is enriched in CD45RBdim mature memory T cells that are potent helpers for B cell differentiation. Arthritis Rheum. (1992) 35:1455–65. 10.1002/art.1780351209 1472123

[90] Klarenbeek PL de Hair MJH Doorenspleet ME van Schaik BDC Esveldt REE van de Sande MGH. Inflamed target tissue provides a specific niche for highly expanded T-cell clones in early human autoimmune disease. Ann Rheum Dis. (2012) 71:1088–93. 10.1136/annrheumdis-2011-200612 22294635

[91] Kremer JM Westhovens R Leon M Di Giorgio E Alten R Steinfeld S. Treatment of rheumatoid arthritis by selective inhibition of T-cell activation with fusion protein CTLA4Ig. N Engl J Med. (2003) 349:1907–15. 10.1056/NEJMoa035075 14614165

[92] Gregersen PK Silver J Winchester RJ. The shared epitope hypothesis. An approach to understanding the molecular genetics of susceptibility to rheumatoid arthritis. Arthritis Rheum. (1987) 30:1205–13. 10.1002/art.1780301102 2446635

[93] Dolhain RJ van der Heiden AN ter Haar NT Breedveld FC Miltenburg AM. Shift toward T lymphocytes with a T helper 1 cytokine-secretion profile in the joints of patients with rheumatoid arthritis. Arthritis Rheum. (1996) 39:1961–9. 8961900 10.1002/art.1780391204

[94] Miltenburg AM van Laar JM de Kuiper R Daha MR Breedveld FC. T cells cloned from human rheumatoid synovial membrane functionally represent the Th1 subset. Scand J Immunol. (1992) 35:603–10. 10.1111/j.1365-3083.1992.tb03260.x 1349769

[95] Patel DD Zachariah JP Whichard LP. CXCR3 and CCR5 ligands in rheumatoid arthritis synovium. Clin Immunol. (2001) 98:39–45. 10.1006/clim.2000.4957 11141325

[96] Sallusto F Lenig D Mackay CR Lanzavecchia A. Flexible programs of chemokine receptor expression on human polarized T helper 1 and 2 lymphocytes. J Exp Med. (1998) 187:875–83. 10.1084/jem.187.6.875 9500790 PMC2212187

[97] Qin S Rottman JB Myers P Kassam N Weinblatt M Loetscher M. The chemokine receptors CXCR3 and CCR5 mark subsets of T cells associated with certain inflammatory reactions. J Clin Invest. (1998) 101:746–54. 10.1172/JCI1422 9466968 PMC508621

[98] Loetscher M Gerber B Loetscher P Jones SA Piali L Clark-Lewis I. Chemokine receptor specific for IP10 and mig: structure, function, and expression in activated T-lymphocytes. J Exp Med. (1996) 184:963–9. 10.1084/jem.184.3.963 9064356 PMC2192763

[99] Maruotti N Cantatore FP Crivellato E Vacca A Ribatti D. Macrophages in rheumatoid arthritis. Histol Histopathol. (2007) 22:581–6. 10.14670/HH-22.581 17330813

[100] Gracie JA Forsey RJ Chan WL Gilmour A Leung BP Greer MR. A proinflammatory role for IL-18 in rheumatoid arthritis. J Clin Invest. (1999) 104:1393–401. 10.1172/JCI7317 10562301 PMC409841

[101] Morita Y Yamamura M Nishida K Harada S Okamoto H Inoue H. Expression of interleukin-12 in synovial tissue from patients with rheumatoid arthritis. Arthritis Rheum. (1998) 41:306–14. 9485089 10.1002/1529-0131(199802)41:2<306::AID-ART15>3.0.CO;2-4

[102] Firestein GS Zvaifler NJ. How important are T cells in chronic rheumatoid synovitis? Arthritis Rheum. (1990) 33:768–73. 10.1002/art.1780330602 2194461

[103] Firestein GS Zvaifler NJ Peripheral blood and synovial fluid monocyte activation in inflammatory arthritis. II. Low levels of synovial fluid and synovial tissue interferon suggest that gamma-interferon is not the primary macrophage activating factor. Arthritis Rheum. (1987) 30:864–71. 10.1002/art.1780300804 3115274

[104] Jin W Dong C. IL-17 cytokines in immunity and inflammation. Emerg Microbes Infect. (2013) 2:e60. 10.1038/emi.2013.58 26038490 PMC3820987

[105] Annunziato F Cosmi L Liotta F Maggi E Romagnani S. Human Th1 dichotomy: origin, phenotype and biologic activities. Immunology. (2014). 10.1111/imm.12399. [Epub ahead of print]. 25284714 PMC4557671

[106] Cosmi L Liotta F Maggi E Romagnani S Annunziato F. Th17 and non-classic Th1 cells in chronic inflammatory disorders: two sides of the same coin. Int Arch Allergy Immunol. (2014) 164:171–7. 10.1159/000363502 25033972

[107] Maggi L Santarlasci V Capone M Rossi MC Querci V Mazzoni A. Distinctive features of classic and nonclassic (Th17 derived) human Th1 cells. Eur J Immunol. (2012) 42:3180–8. 10.1002/eji.201242648 22965818

[108] Cosmi L Cimaz R Maggi L Santarlasci V Capone M Borriello F. Evidence of the transient nature of the Th17 phenotype of CD4+CD161+ T cells in the synovial fluid of patients with juvenile idiopathic arthritis. Arthritis Rheum. (2011) 63:2504–15. 10.1002/art.30332 21381000

[109] Geginat J Paroni M Kastirr I Larghi P Pagani M Abrignani S. Reverse plasticity: TGF-beta and IL-6 induce Th1-to-Th17-cell transdifferentiation in the gut. Eur J Immunol. (2016) 46:2306–10. 10.1002/eji.201646618 27726139

[110] Martinez-Sanchez ME Mendoza L Villarreal C Alvarez-Buylla ER. A minimal regulatory network of extrinsic and intrinsic factors recovers observed patterns of CD4+ T cell differentiation and plasticity. PLoS Comput Biol. (2015) 11:e1004324. 10.1371/journal.pcbi.1004324 26090929 PMC4475012

[111] Liu HP Cao AT Feng T Li Q Zhang W Yao S. TGF-beta converts Th1 cells into Th17 cells through stimulation of Runx1 expression. Eur J Immunol. (2015) 45:1010–8. 10.1002/eji.201444726 25605286 PMC4441226

[112] Hirahara K Poholek A Vahedi G Laurence A Kanno Y Milner JD. Mechanisms underlying helper T-cell plasticity: implications for immune-mediated disease. J Allergy Clin Immunol. (2013) 131:1276–87. 10.1016/j.jaci.2013.03.015 23622118 PMC3677748

[113] Kotake S Nanke Y Yago T Kawamoto M Kobashigawa T Yamanaka H. Elevated ratio of Th17 cell-derived Th1 cells (CD161(+)Th1 cells) to CD161(+)Th17 cells in peripheral blood of early-onset rheumatoid arthritis patients. Biomed Res Int. (2016) 2016:4186027. 10.1155/2016/4186027 27123445 PMC4829689

[114] Banuelos J Shin S Cao Y Bochner BS Morales-Nebreda L Budinger GR. BCL-2 protects human and mouse Th17 cells from glucocorticoid-induced apoptosis. Allergy. (2016) 71:640–50. 10.1111/all.12840 26752231 PMC4844778

[115] Zubiaga AM Munoz E Huber BT. IL-4 and IL-2 selectively rescue Th cell subsets from glucocorticoid-induced apoptosis. J Immunol. (1992) 149:107–12. 1535084

[116] Verhoef CM van Roon JA Vianen ME Lafeber FP Bijlsma JW. The immune suppressive effect of dexamethasone in rheumatoid arthritis is accompanied by upregulation of interleukin 10 and by differential changes in interferon gamma and interleukin 4 production. Ann Rheum Dis. (1999) 58:49–54. 10.1136/ard.58.1.49 10343540 PMC1752750

[117] Arya SK Wong-Staal F Gallo RC. Dexamethasone-mediated inhibition of human T cell growth factor and gamma-interferon messenger RNA. J Immunol. (1984) 133:273–6. 6427338

[118] Liberman AC Refojo D Druker J Toscano M Rein T Holsboer F. The activated glucocorticoid receptor inhibits the transcription factor T-bet by direct protein-protein interaction. FASEB J. (2007) 21:1177–88. 10.1096/fj.06-7452com 17215482

[119] Franchimont D Galon J Gadina M Visconti R Zhou Y Aringer M. Inhibition of Th1 immune response by glucocorticoids: dexamethasone selectively inhibits IL-12-induced Stat4 phosphorylation in T lymphocytes. J Immunol. (2000) 164:1768–74. 10.4049/jimmunol.164.4.1768 10657623

[120] de Castro Kroner J Knoke K Kofler DM Steiger J Fabri M. Glucocorticoids promote intrinsic human TH17 differentiation. J Allergy Clin Immunol. (2018) 142:1669–73 e11. 10.1016/j.jaci.2018.07.019 30092286

[121] Kotake S Udagawa N Takahashi N Matsuzaki K Itoh K Ishiyama S. IL-17 in synovial fluids from patients with rheumatoid arthritis is a potent stimulator of osteoclastogenesis. J Clin Invest. (1999) 103:1345–52. 10.1172/JCI5703 10225978 PMC408356

[122] Kaplan MJ. Role of neutrophils in systemic autoimmune diseases. Arthritis Res Ther. (2013) 15:219. 10.1186/ar4325 24286137 PMC3978765

[123] Wright HL Moots RJ Edwards SW. The multifactorial role of neutrophils in rheumatoid arthritis. Nat Rev Rheumatol. (2014) 10:593–601. 10.1038/nrrheum.2014.80 24914698

[124] Liles WC Dale DC Klebanoff SJ. Glucocorticoids inhibit apoptosis of human neutrophils. Blood. (1995) 86:3181–8. 7579413

[125] La Paglia GMC Leone MC Lepri G Vagelli R Valentini E Alunno A. One year in review 2017: systemic lupus erythematosus. Clin Exp Rheumatol. (2017) 35:551–61. Retrieved from: https://www.clinexprheumatol.org/article.asp?a=12006 28721860

[126] Hahn BH. Antibodies to DNA. N Engl J Med. (1998) 338:1359–68. 10.1056/NEJM199805073381906 9571257

[127] Banchereau J Pascual V. Type I interferon in systemic lupus erythematosus and other autoimmune diseases. Immunity. (2006) 25:383–92. 10.1016/j.immuni.2006.08.010 16979570

[128] Guiducci C Gong M Xu Z Gill M Chaussabel D Meeker T. TLR recognition of self nucleic acids hampers glucocorticoid activity in lupus. Nature. (2010) 465:937–41. 10.1038/nature09102 20559388 PMC2964153

[129] Theofilopoulos AN Baccala R Beutler B Kono DH. Type I interferons (alpha/beta) in immunity and autoimmunity. Annu Rev Immunol. (2005) 23:307–36. 10.1146/annurev.immunol.23.021704.115843 15771573

[130] Ronnblom L Pascual V. The innate immune system in SLE: type I interferons and dendritic cells. Lupus. (2008) 17:394–9. 10.1177/0961203308090020 18490415 PMC3694565

[131] Shlomchik MJ Madaio MP Ni D Trounstein M Huszar D. The role of B cells in lpr/lpr-induced autoimmunity. J Exp Med. (1994) 180:1295–306. 10.1084/jem.180.4.1295 7931063 PMC2191708

[132] Chan OT Hannum LG Haberman AM Madaio MP Shlomchik MJ. A novel mouse with B cells but lacking serum antibody reveals an antibody-independent role for B cells in murine lupus. J Exp Med. (1999) 189:1639–48. 10.1084/jem.189.10.1639 10330443 PMC2193634

[133] Lugar PL Love C Grammer AC Dave SS Lipsky PE. Molecular characterization of circulating plasma cells in patients with active systemic lupus erythematosus. PLoS ONE. (2012) 7:e44362. 10.1371/journal.pone.0044362 23028528 PMC3448624

[134] Luo J Niu X Liu H Zhang M Chen M Deng S. Up-regulation of transcription factor Blimp1 in systemic lupus erythematosus. Mol Immunol. (2013) 56:574–82. 10.1016/j.molimm.2013.05.241 23911415

[135] Pers JO Daridon C Devauchelle V Jousse S Saraux A Jamin C. BAFF overexpression is associated with autoantibody production in autoimmune diseases. Ann N Y Acad Sci. (2005) 1050:34–9. 10.1196/annals.1313.004 16014518

[136] Bengtsson AA Ronnblom L. Role of interferons in SLE. Best Pract Res Clin Rheumatol. (2017) 31:415–28. 10.1016/j.berh.2017.10.003 29224681

[137] Merrill JT Neuwelt CM Wallace DJ Shanahan JC Latinis KM Oates JC. Efficacy and safety of rituximab in moderately-to-severely active systemic lupus erythematosus: the randomized, double-blind, phase II/III systemic lupus erythematosus evaluation of rituximab trial. Arthritis Rheum. (2010) 62:222–33. 10.1002/art.27233 20039413 PMC4548300

[138] Scherlinger M Carcaud C Truchetet ME Barnetche T Duffau P Couzi L. Rituximab in moderate to severe non-renal systemic lupus erythematosus: a reanalysis of the EXPLORER study. Ann Rheum Dis. (2019) 78:1007–10. 10.1136/annrheumdis-2018-214833 30610064

[139] Jacobi AM Huang W Wang T Freimuth W Sanz I Furie R. Effect of long-term belimumab treatment on B cells in systemic lupus erythematosus: extension of a phase II, double-blind, placebo-controlled, dose-ranging study. Arthritis Rheum. (2010) 62:201–10. 10.1002/art.27189 20039404 PMC2857977

[140] Dorner T Lipsky PE. Beyond pan-B-cell-directed therapy - new avenues and insights into the pathogenesis of SLE. Nat Rev Rheumatol. (2016) 12:645–57. 10.1038/nrrheum.2016.158 27733759

[141] Yan SX Deng XM Wang QT Sun XJ Wei W. Prednisone treatment inhibits the differentiation of B lymphocytes into plasma cells in MRL/MpSlac-lpr mice. Acta Pharmacol Sin. (2015) 36:1367–76. 10.1038/aps.2015.76 26456588 PMC4635331

[142] Haneda M Owaki M Kuzuya T Iwasaki K Miwa Y Kobayashi T. Comparative analysis of drug action on B-cell proliferation and differentiation for mycophenolic acid, everolimus, and prednisolone. Transplantation. (2014) 97:405–12. 10.1097/01.TP.0000441826.70687.f6 24445924 PMC4032219

[143] Odendahl M Jacobi A Hansen A Feist E Hiepe F Burmester GR. Disturbed peripheral B lymphocyte homeostasis in systemic lupus erythematosus. J Immunol. (2000) 165:5970–9. 10.4049/jimmunol.165.10.5970 11067960

[144] Chatham WW Kimberly RP. Treatment of lupus with corticosteroids. Lupus. (2001) 10:140–7. 10.1191/096120301675075008 11315342

[145] Jiang T Liu S Tan M Huang F Sun Y Dong X. The phase-shift mutation in the glucocorticoid receptor gene: potential etiologic significance of neuroendocrine mechanisms in lupus nephritis. Clin Chim Acta. (2001) 313:113–7. 10.1016/S0009-8981(01)00661-1 11694247

[146] Andreae J Tripmacher R Weltrich R Rohde W Keitzer R Wahn U. Effect of glucocorticoid therapy on glucocorticoid receptors in children with autoimmune diseases. Pediatr Res. (2001) 49:130–5. 10.1203/00006450-200101000-00025 11134503

[147] Neeck G Kluter A Dotzlaw H Eggert M. Involvement of the glucocorticoid receptor in the pathogenesis of rheumatoid arthritis. Ann N Y Acad Sci. (2002) 966:491–5. 10.1111/j.1749-6632.2002.tb04252.x 12114309

[148] Gladman DD Urowitz MB Doris F Lewandowski K Anhorn K. Glucocorticoid receptors in systemic lupus erythematosus. J Rheumatol. (1991) 18:681–4. 1865413

[149] van Everdingen AA Huisman AM Wenting MJ van Reesema S Jacobs JW Bijlsma JW. Down regulation of glucocorticoid receptors in early-diagnosed rheumatoid arthritis. Clin Exp Rheumatol. (2002) 20:463–8. Retrieved from: https://www.clinexprheumatol.org/article.asp?a=1395 12175100

[150] Tanaka H Akama H Ichikawa Y Makino I Homma M. Glucocorticoid receptor in patients with lupus nephritis: relationship between receptor levels in mononuclear leukocytes and effect of glucocorticoid therapy. J Rheumatol. (1992) 19:878–83. 1404124

[151] Li X Zhang FS Zhang JH Wang JY. Negative relationship between expression of glucocorticoid receptor alpha and disease activity: glucocorticoid treatment of patients with systemic lupus erythematosus. J Rheumatol. (2010) 37:316–21. 10.3899/jrheum.090191 20032106

[152] Jacobi AM Mei H Hoyer BF Mumtaz IM Thiele K Radbruch A. HLA-DRhigh/CD27high plasmablasts indicate active disease in patients with systemic lupus erythematosus. Ann Rheum Dis. (2010) 69:305–8. 10.1136/ard.2008.096495 19196727

[153] Jones SA Toh AE Odobasic D Oudin MA Cheng Q Lee JP. Glucocorticoid-induced leucine zipper (GILZ) inhibits B cell activation in systemic lupus erythematosus. Ann Rheum Dis. (2016) 75:739–47. 10.1136/annrheumdis-2015-207744 26612340

[154] Gao H Wang Q Yu X Liu J Bai S Feng J. Molecular mechanisms of glucocorticoid resistance in systemic lupus erythematosus: a review. Life Sci. (2018) 209:383–7. 10.1016/j.lfs.2018.08.038 30125579

[155] Beck WT Grogan TM Willman CL Cordon-Cardo C Parham DM Kuttesch JF. Methods to detect P-glycoprotein-associated multidrug resistance in patients' tumors: consensus recommendations. Cancer Res. (1996) 56:3010–20. 8674056

[156] Picchianti-Diamanti A Rosado MM Scarsella M Laganà B Amelio R. P-glycoprotein and drug resistance in systemic autoimmune diseases. Int J Mol Sci. (2014) 15:4965–76. 10.3390/ijms15034965 24658440 PMC3975434

[157] Perez-Guerrero EE Gamez-Nava JI Munoz-Valle JF Cardona-Munoz EG Bonilla-Lara D Fajardo-Robledo NS. Serum levels of P-glycoprotein and persistence of disease activity despite treatment in patients with systemic lupus erythematosus. Clin Exp Med. (2018) 18:109–17. 10.1007/s10238-017-0459-0 28243944

[158] Kansal A Tripathi D Rai MK Agarwal V. Persistent expression and function of P-glycoprotein on peripheral blood lymphocytes identifies corticosteroid resistance in patients with systemic lupus erythematosus. Clin Rheumatol. (2016) 35:341–9. 10.1007/s10067-015-3079-7 26415739

[159] Santos LL Morand EF. Macrophage migration inhibitory factor: a key cytokine in RA, SLE and atherosclerosis. Clin Chim Acta. (2009) 399:1–7. 10.1016/j.cca.2008.09.014 18838066

[160] Foote A Briganti EM Kipen Y Santos L Leech M Morand EF. Macrophage migration inhibitory factor in systemic lupus erythematosus. J Rheumatol. (2004) 31:268–73. Retrieved from: http://www.jrheum.org/content/31/2/268 14760795

[161] Berdeli A Mir S Ozkayin N Serdaroglu E Tabel Y Cura A. Association of macrophage migration inhibitory factor −173C allele polymorphism with steroid resistance in children with nephrotic syndrome. Pediatr Nephrol. (2005) 20:1566–71. 10.1007/s00467-005-1930-9 16133063

[162] Wang F-F Zhu L-A Zou Y-Q Zheng H Wilson A Yang C-D. New insights into the role and mechanism of macrophage migration inhibitory factor in steroid-resistant patients with systemic lupus erythematosus. Arthritis Res Ther. (2012) 14:R103. 10.1186/ar3828 22551315 PMC3446480

[163] Ruiz-Arruza I Ugarte A Cabezas-Rodriguez I Medina JA Moran MA Ruiz-Irastorza G. Glucocorticoids and irreversible damage in patients with systemic lupus erythematosus. Rheumatology. (2014) 53:1470–6. 10.1093/rheumatology/keu148 24681836

[164] Moallem E Koren E Ulmansky R Pizov G Barlev M Barenholz Y. A liposomal steroid nano-drug for treating systemic lupus erythematosus. Lupus. (2016) 25:1209–16. 10.1177/0961203316636468 26957351

[165] Avnir Y Turjeman K Tulchinsky D Sigal A Kizelsztein P Tzemach D. Fabrication principles and their contribution to the superior *in vivo* therapeutic efficacy of nano-liposomes remote loaded with glucocorticoids. PLoS ONE. (2011) 6:e25721. 10.1371/journal.pone.0025721 21998684 PMC3188566

[166] Avnir Y Ulmansky R Wasserman V Even-Chen S Broyer M Barenholz Y. Amphipathic weak acid glucocorticoid prodrugs remote-loaded into sterically stabilized nanoliposomes evaluated in arthritic rats and in a Beagle dog: a novel approach to treating autoimmune arthritis. Arthritis Rheum. (2008) 58:119–29. 10.1002/art.23230 18163482

[167] Turjeman K Bavli Y Kizelsztein P Schilt Y Allon N Katzir TB. Nano-drugs based on nano sterically stabilized liposomes for the treatment of inflammatory neurodegenerative diseases. PLoS ONE. (2015) 10:e0130442. 10.1371/journal.pone.0130442 26147975 PMC4492950

[168] Ulmansky R Turjeman K Baru M Katzavian G Harel M Sigal A. Glucocorticoids in nano-liposomes administered intravenously and subcutaneously to adjuvant arthritis rats are superior to the free drugs in suppressing arthritis and inflammatory cytokines. J Control Release. (2012) 160:299–305. 10.1016/j.jconrel.2011.12.024 22226777

[169] Waknine-Grinberg JH Even-Chen S Avichzer J Turjeman K Bentura-Marciano A Haynes RK. Glucocorticosteroids in nano-sterically stabilized liposomes are efficacious for elimination of the acute symptoms of experimental cerebral malaria. PLoS ONE. (2013) 8:e72722. 10.1371/journal.pone.0072722 23991146 PMC3753236

[170] van der Geest T Metselaar JM Gerrits D van Lent PL Storm G Laverman P. [^(18)^]F FDG PET/CT imaging to monitor the therapeutic effect of liposome-encapsulated prednisolone in experimental rheumatoid arthritis. J Control Release. (2015) 209:20–6. 10.1016/j.jconrel.2015.04.019 25902038

[171] Anderson GP Coyle AJ. TH2 and ‘TH2-like' cells in allergy and asthma: pharmacological perspectives. Trends Pharmacol Sci. (1994) 15:324–32. 10.1016/0165-6147(94)90027-2 7992386

[172] Banuelos J Lu NZ. A gradient of glucocorticoid sensitivity among helper T cell cytokines. Cytokine Growth Factor Rev. (2016) 31:27–35. 10.1016/j.cytogfr.2016.05.002 27235091 PMC5050075

[173] Anthony RM Rutitzky LI Urban JF Jr Stadecker MJ Gause WC. Protective immune mechanisms in helminth infection. Nat Rev Immunol. (2007) 7:975–87. 10.1038/nri2199 18007680 PMC2258092

[174] Allen JE Maizels RM. Diversity and dialogue in immunity to helminths. Nat Rev Immunol. (2011) 11:375–88. 10.1038/nri2992 21610741

[175] Hermann-Kunz E Allergische Krankheiten in DeutschlandErgebnisse einer repräsentativen Studie. Bundesgesundheitsblatt. (2000) 43:400–6. 10.1007/s001030070045

[176] Schmitz R Atzpodien K Schlaud M. Prevalence and risk factors of atopic diseases in German children and adolescents. Pediatr Allergy Immunol. (2012) 23:716–23. 10.1111/j.1399-3038.2012.01342.x 22882467

[177] Muehling LM Lawrence MG Woodfolk JA. Pathogenic CD4^(+)^ T cells in patients with asthma. J Allergy Clin Immunol. (2017) 140:1523–40. 10.1016/j.jaci.2017.02.025 28442213 PMC5651193

[178] Wills-Karp M. Interleukin-13 in asthma pathogenesis. Immunol Rev. (2004) 202:175–90. 10.1111/j.0105-2896.2004.00215.x 15546393

[179] Cohn L Elias JA Chupp GL. Asthma: mechanisms of disease persistence and progression. Annu Rev Immunol. (2004) 22:789–815. 10.1146/annurev.immunol.22.012703.104716 15032597

[180] Umetsu DT DeKruyff RH. The regulation of allergy and asthma. Immunol Rev. (2006) 212:238–55. 10.1111/j.0105-2896.2006.00413.x 16903918

[181] Klimek L Hogger P Pfaar O. [Mechanism of action of nasal glucocorticosteroids in the treatment of allergic rhinitis. Part 1: Pathophysiology, molecular basis]. Hno. (2012) 60:611–7. 10.1007/s00106-012-2483-4 22532281

[182] Barnes PJ. Th2 cytokines and asthma: an introduction. Respir Res. (2001) 2:64–5. 10.1186/rr39 11686866 PMC59569

[183] Wills-Karp M. The gene encoding interleukin-13: a susceptibility locus for asthma and related traits. Respir Res. (2000) 1:19–23. 10.1186/rr7 11667960 PMC59537

[184] Clutterbuck EJ Hirst EM Sanderson CJ. Human interleukin-5 (IL-5) regulates the production of eosinophils in human bone marrow cultures: comparison and interaction with IL-1, IL-3, IL-6, and GMCSF. Blood. (1989) 73:1504–12. 2653458

[185] Ochiai K Kagami M Matsumura R Tomioka H IL-5 but not interferon-gamma (IFN-gamma) inhibits eosinophil apoptosis by up-regulation of bcl-2 expression. Clin Exp Immunol. (1997) 107:198–204. 10.1046/j.1365-2249.1997.d01-884.x 9010276 PMC1904544

[186] Hamid Q Boguniewicz M Leung DY. Differential *in situ* cytokine gene expression in acute versus chronic atopic dermatitis. J Clin Invest. (1994) 94:870–6. 10.1172/JCI117408 8040343 PMC296169

[187] Benson M Strannegard IL Strannegard O Wennergren G Topical steroid treatment of allergic rhinitis decreases nasal fluid TH2 cytokines, eosinophils, eosinophil cationic protein, and IgE but has no significant effect on IFN-gamma, IL-1beta, TNF-alpha, or neutrophils. J Allergy Clin Immunol. (2000) 106:307–12. 10.1067/mai.2000.108111 10932075

[188] Hamid Q Azzawi M Ying S Moqbel R Wardlaw AJ Corrigan CJ. Expression of mRNA for interleukin-5 in mucosal bronchial biopsies from asthma. J Clin Invest. (1991) 87:1541–6. 10.1172/JCI115166 2022726 PMC295235

[189] Robinson DS Hamid Q Ying S Tsicopoulos A Barkans J Bentley AM. Predominant TH2-like bronchoalveolar T-lymphocyte population in atopic asthma. N Engl J Med. (1992) 326:298–304. 10.1056/NEJM199201303260504 1530827

[190] Ying S Durham SR Corrigan CJ Hamid Q Kay AB. Phenotype of cells expressing mRNA for TH2-type (interleukin 4 and interleukin 5) and TH1-type (interleukin 2 and interferon gamma) cytokines in bronchoalveolar lavage and bronchial biopsies from atopic asthmatic and normal control subjects. Am J Respir Cell Mol Biol. (1995) 12:477–87. 10.1165/ajrcmb.12.5.7742012 7742012

[191] Walker C Bode E Boer L Hansel TT Blaser K Virchow JC Jr. Allergic and nonallergic asthmatics have distinct patterns of T-cell activation and cytokine production in peripheral blood and bronchoalveolar lavage. Am Rev Respir Dis. (1992) 146:109–15. 10.1164/ajrccm/146.1.109 1626792

[192] von Bernus L Hogger P Pfaar O Klimek L. [Mechanism of action of nasal glucocorticosteroids in the treatment of allergic rhinitis. Part 2: practical aspects of application]. Hno. (2012) 60:700–6. 10.1007/s00106-012-2484-3 22532282

[193] Bentley AM Hamid Q Robinson DS Schotman E Meng Q Assoufi B. Prednisolone treatment in asthma. Reduction in the numbers of eosinophils, T cells, tryptase-only positive mast cells, and modulation of IL-4, IL-5, and interferon-gamma cytokine gene expression within the bronchial mucosa. Am J Respir Crit Care Med. (1996) 153:551–6. 10.1164/ajrccm.153.2.8564096 8564096

[194] Rak S Jacobson MR Sudderick RM Masuyama K Juliusson S Kay AB. Influence of prolonged treatment with topical corticosteroid (fluticasone propionate) on early and late phase nasal responses and cellular infiltration in the nasal mucosa after allergen challenge. Clin Exp Allergy. (1994) 24:930–9. 10.1111/j.1365-2222.1994.tb02724.x 7842362

[195] Daynes RA Araneo BA. Contrasting effects of glucocorticoids on the capacity of T cells to produce the growth factors interleukin 2 and interleukin 4. Eur J Immunol. (1989) 19:2319–25. 10.1002/eji.1830191221 2606141

[196] Daynes RA Araneo BA Dowell TA Huang K Dudley D. Regulation of murine lymphokine production *in vivo*. III. The lymphoid tissue microenvironment exerts regulatory influences over T helper cell function. J Exp Med. (1990) 171:979–96. 10.1084/jem.171.4.979 2139106 PMC2187824

[197] Moynihan JA Karp JD Cohen N Cocke R. Alterations in interleukin-4 and antibody production following pheromone exposure: role of glucocorticoids. J Neuroimmunol. (1994) 54:51–8. 10.1016/0165-5728(94)90230-5 7929803

[198] Miyaura H Iwata M. Direct and indirect inhibition of Th1 development by progesterone and glucocorticoids. J Immunol. (2002) 168:1087–94. 10.4049/jimmunol.168.3.1087 11801642

[199] Franchimont D Louis E Dewe W Martens H Vrindts-Gevaert Y De Groote D. Effects of dexamethasone on the profile of cytokine secretion in human whole blood cell cultures. Regul Pept. (1998) 73:59–65. 10.1016/S0167-0115(97)01063-X 9537674

[200] Agarwal SK Marshall GD Jr. Glucocorticoid-induced type 1/type 2 cytokine alterations in humans: a model for stress-related immune dysfunction. J Interferon Cytokine Res. (1998) 18:1059–68. 10.1089/jir.1998.18.1059 9877450

[201] Ramirez F. Glucocorticoids induce a Th2 response *in vitro*. Dev Immunol. (1998) 6:233–43. 10.1155/1998/73401 9814597 PMC2276017

[202] Mozo L Gayo A Suarez A Rivas D Zamorano J Gutierrez C. Glucocorticoids inhibit IL-4 and mitogen-induced IL-4R alpha chain expression by different posttranscriptional mechanisms. J Allergy Clin Immunol. (1998) 102(6 Pt 1):968–76. 10.1016/S0091-6749(98)70335-5 9847438

[203] Wu CY Fargeas C Nakajima T Delespesse G. Glucocorticoids suppress the production of interleukin 4 by human lymphocytes. Eur J Immunol. (1991) 21:2645–7. 10.1002/eji.1830211053 1915563

[204] Braun CM Huang SK Bashian GG Kagey-Sobotka A Lichtenstein LM Essayan DM. Corticosteroid modulation of human, antigen-specific Th1 and Th2 responses. J Allergy Clin Immunol. (1997) 100:400–7. 10.1016/S0091-6749(97)70255-0 9314354

[205] Beck IM Van Crombruggen K Holtappels G Daubeuf F Frossard N Bachert C. Differential cytokine profiles upon comparing selective versus classic glucocorticoid receptor modulation in human peripheral blood mononuclear cells and inferior turbinate tissue. PLoS ONE. (2015) 10:e0123068. 10.1371/journal.pone.0123068 25875480 PMC4395417

[206] Kovalovsky D Refojo D Holsboer F Arzt E. Molecular mechanisms and Th1/Th2 pathways in corticosteroid regulation of cytokine production. J Neuroimmunol. (2000) 109:23–9. 10.1016/S0165-5728(00)00298-8 10969177

[207] Refojo D Liberman AC Giacomini D Carbia Nagashima A Graciarena M Echenique C. Integrating systemic information at the molecular level: cross-talk between steroid receptors and cytokine signaling on different target cells. Ann N Y Acad Sci. (2003) 992:196–204. 10.1111/j.1749-6632.2003.tb03150.x 12794059

[208] Liberman AC Refojo D Arzt E. Cytokine signaling/transcription factor cross-talk in T cell activation and Th1-Th2 differentiation. Arch Immunol Ther Exp. (2003) 51:351–65. Retrieved from: https://pdfs.semanticscholar.org/625d/334aa073fd452518ef26c73a7d39431d0ffa.pdf 14692657

[209] Jee YK Gilmour J Kelly A Bowen H Richards D Soh C. Repression of interleukin-5 transcription by the glucocorticoid receptor targets GATA3 signaling and involves histone deacetylase recruitment. J Biol Chem. (2005) 280:23243–50. 10.1074/jbc.M503659200 15826950

[210] Li-Weber M Krammer PH. Regulation of IL4 gene expression by T cells and therapeutic perspectives. Nat Rev Immunol. (2003) 3:534–43. 10.1038/nri1128 12876556

[211] Maneechotesuwan K Yao X Ito K Jazrawi E Usmani OS Adcock IM. Suppression of GATA-3 nuclear import and phosphorylation: a novel mechanism of corticosteroid action in allergic disease. PLoS Med. (2009) 6:e1000076. 10.1371/journal.pmed.1000076 19436703 PMC2674207

[212] Hu C Li Z Feng J Tang Y Qin L Hu X. Glucocorticoids modulate Th1 and Th2 responses in asthmatic mouse models by inhibition of Notch1 signaling. Int Arch Allergy Immunol. (2018) 175:44–52. 10.1159/000485890 29342458

[213] von Hertzen LC. Maternal stress and T-cell differentiation of the developing immune system: possible implications for the development of asthma and atopy. J Allergy Clin Immunol. (2002) 109:923–8. 10.1067/mai.2002.124776 12063519

[214] Miyasaka T Dobashi-Okuyama K Takahashi T Takayanagi M Ohno I. The interplay between neuroendocrine activity and psychological stress-induced exacerbation of allergic asthma. Allergol Int. (2018) 67:32–42. 10.1016/j.alit.2017.04.013 28539203

[215] Hussain Z Katas H Mohd Amin MC Kumolosasi E Sahudin S. Downregulation of immunological mediators in 2,4-dinitrofluorobenzene-induced atopic dermatitis-like skin lesions by hydrocortisone-loaded chitosan nanoparticles. Int J Nanomed. (2014) 9:5143–56. 10.2147/IJN.S71543 25395851 PMC4227626

[216] Erin EM Zacharasiewicz AS Nicholson GC Tan AJ Higgins LA Williams TJ. Topical corticosteroid inhibits interleukin-4,−5 and−13 in nasal secretions following allergen challenge. Clin Exp Allergy. (2005) 35:1608–14. 10.1111/j.1365-2222.2005.02381.x 16393327

[217] Bradding P Feather IH Wilson S Holgate ST Howarth PH. Cytokine immunoreactivity in seasonal rhinitis: regulation by a topical corticosteroid. Am J Respir Crit Care Med. (1995) 151:1900–6. 10.1164/ajrccm.151.6.7767538 7767538

[218] Wen W Liu W Zhang L Bai J Fan Y Xia W. Increased neutrophilia in nasal polyps reduces the response to oral corticosteroid therapy. J Allergy Clin Immunol. (2012) 129:1522–8.e5. 10.1016/j.jaci.2012.01.079 22460066

[219] Naseer T Minshall EM Leung DY Laberge S Ernst P Martin RJ. Expression of IL-12 and IL-13 mRNA in asthma and their modulation in response to steroid therapy. Am J Respir Crit Care Med. (1997) 155:845–51. 10.1164/ajrccm.155.3.9117015 9117015

[220] Gemou-Engesaeth V Bush A Kay AB Hamid Q Corrigan CJ. Inhaled glucocorticoid therapy of childhood asthma is associated with reduced peripheral blood T cell activation and ‘Th2-type' cytokine mRNA expression. Pediatrics. (1997) 99:695–703. 10.1542/peds.99.5.695 9113946

[221] Robinson D Hamid Q Ying S Bentley A Assoufi B Durham S. Prednisolone treatment in asthma is associated with modulation of bronchoalveolar lavage cell interleukin-4, interleukin-5, and interferon-gamma cytokine gene expression. Am Rev Respir Dis. (1993) 148:401–6. 10.1164/ajrccm/148.2.401 8342904

[222] Smith SJ Piliponsky AM Rosenhead F Elchalal U Nagler A Levi-Schaffer F. Dexamethasone inhibits maturation, cytokine production and Fc epsilon RI expression of human cord blood-derived mast cells. Clin Exp Allergy. (2002) 32:906–13. 10.1046/j.1365-2745.2002.01418.x 12047438

[223] Crocker IC Zhou CY Bewtra AK Kreutner W Townley RG. Glucocorticosteroids inhibit leukotriene production. Ann Allergy Asthma Immunol. (1997) 78:497–505. 10.1016/S1081-1206(10)63238-3 9164364

[224] Heiman AS Crews FT. Hydrocortisone selectively inhibits IgE-dependent arachidonic acid release from rat peritoneal mast cells. Prostaglandins. (1984) 27:335–43. 10.1016/0090-6980(84)90084-4 6201957

[225] Rider LG Hirasawa N Santini F Beaven MA. Activation of the mitogen-activated protein kinase cascade is suppressed by low concentrations of dexamethasone in mast cells. J Immunol. (1996) 157:2374–80. 8805635

[226] Eklund KK Humphries DE Xia Z Ghildyal N Friend DS Gross V. Glucocorticoids inhibit the cytokine-induced proliferation of mast cells, the high affinity IgE receptor-mediated expression of TNF-alpha, and the IL-10-induced expression of chymases. J Immunol. (1997) 158:4373–80. 9127001

[227] Finotto S Mekori YA Metcalfe DD. Glucocorticoids decrease tissue mast cell number by reducing the production of the c-kit ligand, stem cell factor, by resident cells: *in vitro* and *in vivo* evidence in murine systems. J Clin Invest. (1997) 99:1721–8. 10.1172/JCI119336 9120017 PMC507993

[228] Stellato C Atsuta J Bickel CA Schleimer RP. An *in vitro* comparison of commonly used topical glucocorticoid preparations. J Allergy Clin Immunol. (1999) 104(3 Pt 1):623–9. 10.1016/S0091-6749(99)70334-9 10482838

[229] Meagher LC Cousin JM Seckl JR Haslett C. Opposing effects of glucocorticoids on the rate of apoptosis in neutrophilic and eosinophilic granulocytes. J Immunol. (1996) 156:4422–8. 8666816

[230] Till SJ Jacobson MR O'Brien F Durham SR KleinJan A Fokkens WJ. Recruitment of CD1a+ Langerhans cells to the nasal mucosa in seasonal allergic rhinitis and effects of topical corticosteroid therapy. Allergy. (2001) 56:126–31. 10.1034/j.1398-9995.2001.056002126.x 11167372

[231] Durham SR Gould HJ Thienes CP Jacobson MR Masuyama K Rak S. Expression of epsilon germ-line gene transcripts and mRNA for the epsilon heavy chain of IgE in nasal B cells and the effects of topical corticosteroid. Eur J Immunol. (1997) 27:2899–906. 10.1002/eji.1830271123 9394816

[232] Matsui K Tamai S Ikeda R Betamethasone, but not tacrolimus, suppresses the development of Th2 cells mediated by langerhans cell-like dendritic cells. Biol Pharm Bull. (2016) 39:1220–3. 10.1248/bpb.b16-00075 27374298

[233] Klassen C Karabinskaya A Dejager L Vettorazzi S Van Moorleghem J Luhder F. Airway epithelial cells are crucial targets of glucocorticoids in a mouse model of allergic asthma. J Immunol. (2017) 199:48–61. 10.4049/jimmunol.1601691 28515280

[234] Pawankar R Hayashi M Yamanishi S Igarashi T. The paradigm of cytokine networks in allergic airway inflammation. Curr Opin Allergy Clin Immunol. (2015) 15:41–8. 10.1097/ACI.0000000000000129 25479317

[235] Hawrylowicz CM O'Garra A. Potential role of interleukin-10-secreting regulatory T cells in allergy and asthma. Nat Rev Immunol. (2005) 5:271–83. 10.1038/nri1589 15775993

[236] Gupta A Dimeloe S Richards DF Chambers ES Black C Urry Z. Defective IL-10 expression and *in vitro* steroid-induced IL-17A in paediatric severe therapy-resistant asthma. Thorax. (2014) 69:508–15. 10.1136/thoraxjnl-2013-203421 24347461

[237] John M Lim S Seybold J Jose P Robichaud A O'Connor B. Inhaled corticosteroids increase interleukin-10 but reduce macrophage inflammatory protein-1alpha, granulocyte-macrophage colony-stimulating factor, and interferon-gamma release from alveolar macrophages in asthma. Am J Respir Crit Care Med. (1998) 157:256–62. 10.1164/ajrccm.157.1.9703079 9445307

[238] Borish L Aarons A Rumbyrt J Cvietusa P Negri J Wenzel S. Interleukin-10 regulation in normal subjects and patients with asthma. J Allergy Clin Immunol. (1996) 97:1288–96. 10.1016/S0091-6749(96)70197-5 8648025

[239] Lim S Crawley E Woo P Barnes PJ. Haplotype associated with low interleukin-10 production in patients with severe asthma. Lancet. (1998) 352:113. 10.1016/S0140-6736(98)85018-6 9672280

[240] van Oosterhout AJ Bloksma N. Regulatory T-lymphocytes in asthma. Eur Respir J. (2005) 26:918–32. 10.1183/09031936.05.00011205 16264056

[241] Baatjes AJ Smith SG Watson R Howie K Murphy D Larche M. T regulatory cell phenotypes in peripheral blood and bronchoalveolar lavage from non-asthmatic and asthmatic subjects. Clin Exp Allergy. (2015) 45:1654–62. 10.1111/cea.12594 26177872

[242] Grindebacke H Wing K Andersson AC Suri-Payer E Rak S Rudin A. Defective suppression of Th2 cytokines by CD4CD25 regulatory T cells in birch allergics during birch pollen season. Clin Exp Allergy. (2004) 34:1364–72. 10.1111/j.1365-2222.2004.02067.x 15347368

[243] Xystrakis E Kusumakar S Boswell S Peek E Urry Z Richards DF. Reversing the defective induction of IL-10-secreting regulatory T cells in glucocorticoid-resistant asthma patients. J Clin Invest. (2006) 116:146–55. 10.1172/JCI21759 16341266 PMC1307558

[244] Dao Nguyen X Robinson DS. Fluticasone propionate increases CD4CD25 T regulatory cell suppression of allergen-stimulated CD4CD25 T cells by an IL-10-dependent mechanism. J Allergy Clin Immunol. (2004) 114:296–301. 10.1016/j.jaci.2004.04.048 15316506

[245] Hartl D Koller B Mehlhorn AT Reinhardt D Nicolai T Schendel DJ. Quantitative and functional impairment of pulmonary CD4+CD25hi regulatory T cells in pediatric asthma. J Allergy Clin Immunol. (2007) 119:1258–66. 10.1016/j.jaci.2007.02.023 17412402

[246] Karagiannidis C Akdis M Holopainen P Woolley NJ Hense G Ruckert B. Glucocorticoids upregulate FOXP3 expression and regulatory T cells in asthma. J Allergy Clin Immunol. (2004) 114:1425–33. 10.1016/j.jaci.2004.07.014 15577848

[247] Lin KT Wang LH. New dimension of glucocorticoids in cancer treatment. Steroids. (2016) 111:84–8. 10.1016/j.steroids.2016.02.019 26930575

[248] Schug SA Chandrasena C. Pain management of the cancer patient. Expert Opin Pharmacother. (2015) 16:5–15. 10.1517/14656566.2015.980723 25479712

[249] Leppert W Buss T. The role of corticosteroids in the treatment of pain in cancer patients. Curr Pain Headache Rep. (2012) 16:307–13. 10.1007/s11916-012-0273-z 22644902 PMC3395343

[250] Mitra R Jones S. Adjuvant analgesics in cancer pain: a review. Am J Hosp Palliat Care. (2012) 29:70–9. 10.1177/1049909111413256 21712306

[251] Siegel R DeSantis C Virgo K Stein K Mariotto A Smith T. Cancer treatment and survivorship statistics, 2012. CA Cancer J Clin. (2012) 62:220–41. 10.3322/caac.21149 22700443

[252] Burger JA Montserrat E. Coming full circle: 70 years of chronic lymphocytic leukemia cell redistribution, from glucocorticoids to inhibitors of B-cell receptor signaling. Blood. (2013) 121:1501–9. 10.1182/blood-2012-08-452607 23264597 PMC4968370

[253] Gomes LC Ferrao ALM Evangelista FCG de Almeida TD Barbosa RC Carvalho MDG. Advances in chronic lymphocytic leukemia pharmacotherapy. Biomed Pharmacother. (2018) 97:349–58. 10.1016/j.biopha.2017.10.105 29091884

[254] Shah DS Kumar R. Steroid resistance in leukemia. World J Exp Med. (2013) 3:21–5. 10.5493/wjem.v3.i2.21 24520542 PMC3905587

[255] Dougherty TF White A. Influence of adrenal cortical secretion on blood elements. Science. (1943) 98:367–9. 10.1126/science.98.2547.367 17748288

[256] Parrillo JE Fauci AS. Mechanisms of glucocorticoid action on immune processes. Annu Rev Pharmacol Toxicol. (1979) 19:179–201. 10.1146/annurev.pa.19.040179.001143 222199

[257] Pearson OH Eliel LP Rawson RW Dobriner K Rhodes CP. Adrenocorticotropic hormone- and cortisone-induced regression of lymphoid tumors in man; a preliminary report. Cancer. (1949) 2:943–5. 10.1002/1097-0142(194911)2:6<943::aid-cncr2820020602>3.0.co;2-p 15395192

[258] Shaw RK Boggs DR Silberman HR Frei E A study of prednisone therapy in chronic lymphocytic leukemia. Blood. (1961) 17:182–95.

[259] Manzoni D Catallo R Chebel A Baseggio L Michallet AS Roualdes O. The ibrutinib B-cell proliferation inhibition is potentiated *in vitro* by dexamethasone: application to chronic lymphocytic leukemia. Leuk Res. (2016) 47:1–7. 10.1016/j.leukres.2016.05.003 27235717

[260] Hehlmann R Hochhaus A Baccarani M European L. Chronic myeloid leukaemia. Lancet. (2007) 370:342–50. 10.1016/S0140-6736(07)61165-9 17662883

[261] Radich JP Mauro MJ. Tyrosine kinase inhibitor treatment for newly diagnosed chronic myeloid leukemia. Hematol Oncol Clin North Am. (2017) 31:577–87. 10.1016/j.hoc.2017.04.006 28673389

[262] Yang D Zhang X Zhang X Xu Y. The progress and current status of immunotherapy in acute myeloid leukemia. Ann Hematol. (2017) 96:1965–82. 10.1007/s00277-017-3148-x 29080982

[263] Lohse I Statz-Geary K Brothers SP Wahlestedt C. Precision medicine in the treatment stratification of AML patients: challenges and progress. Oncotarget. (2018) 9:37790–7. 10.18632/oncotarget.26492 30701032 PMC6340870

[264] Hicsonmez G. The effect of steroid on myeloid leukemic cells: the potential of short-course high-dose methylprednisolone treatment in inducing differentiation, apoptosis and in stimulating myelopoiesis. Leuk Res. (2006) 30:60–8. 10.1016/j.leukres.2005.05.015 15979702

[265] Klein K Haarman EG de Haas V Zwaan Ch M Creutzig U Kaspers GL. Glucocorticoid-induced proliferation in untreated pediatric acute myeloid leukemic blasts. Pediatr Blood Cancer. (2016) 63:1457–60. 10.1002/pbc.26011 27093190

[266] Inaba H Pui CH. Glucocorticoid use in acute lymphoblastic leukaemia. Lancet Oncol. (2010) 11:1096–106. 10.1016/S1470-2045(10)70114-5 20947430 PMC3309707

[267] Pui CH Relling MV Downing JR. Acute lymphoblastic leukemia. N Engl J Med. (2004) 350:1535–48. 10.1056/NEJMra023001 15071128

[268] Hanahan D Weinberg RA. Hallmarks of cancer: the next generation. Cell. (2011) 144:646–74. 10.1016/j.cell.2011.02.013 21376230

[269] Jing D Bhadri VA Beck D Thoms JA Yakob NA Wong JW. Opposing regulation of BIM and BCL2 controls glucocorticoid-induced apoptosis of pediatric acute lymphoblastic leukemia cells. Blood. (2015) 125:273–83. 10.1182/blood-2014-05-576470 25336632

[270] Kruth KA Fang M Shelton DN Abu-Halawa O Mahling R Yang H. Suppression of B-cell development genes is key to glucocorticoid efficacy in treatment of acute lymphoblastic leukemia. Blood. (2017) 129:3000–8. 10.1182/blood-2017-02-766204 28424165 PMC5454339

[271] Chen DW Saha V Liu JZ Schwartz JM Krstic-Demonacos M. Erg and AP-1 as determinants of glucocorticoid response in acute lymphoblastic leukemia. Oncogene. (2013) 32:3039–48. 10.1038/onc.2012.321 22869147

[272] Heidari N Miller AV Hicks MA Marking CB Harada H. Glucocorticoid-mediated BIM induction and apoptosis are regulated by Runx2 and c-Jun in leukemia cells. Cell Death Dis. (2012) 3:e349. 10.1038/cddis.2012.89 22825467 PMC3406588

[273] Rainer J Ploner C Jesacher S Ploner A Eduardoff M Mansha M. Glucocorticoid-regulated microRNAs and mirtrons in acute lymphoblastic leukemia. Leukemia. (2009) 23:746–52. 10.1038/leu.2008.370 19148136

